# The Association of the Polymorphisms in the *FUT8*-Related Locus with the Plasma Glycosylation in Post-Traumatic Stress Disorder

**DOI:** 10.3390/ijms24065706

**Published:** 2023-03-16

**Authors:** Lucija Tudor, Gordana Nedic Erjavec, Matea Nikolac Perkovic, Marcela Konjevod, Suzana Uzun, Oliver Kozumplik, Ninoslav Mimica, Gordan Lauc, Dubravka Svob Strac, Nela Pivac

**Affiliations:** 1 Laboratory for Molecular Neuropsychiatry, Division of Molecular Medicine, Rudjer Boskovic Institute, 10000 Zagreb, Croatia; lucija.tudor@irb.hr (L.T.); gordana.nedic.erjavec@irb.hr (G.N.E.); matea.nikolac.perkovic@irb.hr (M.N.P.); marcela.konjevod@irb.hr (M.K.); 2 Department for Biological Psychiatry and Psychogeriatrics, University Hospital Vrapce, 10000 Zagreb, Croatia; suzana.uzun@gmail.com (S.U.); okozumplik@hotmail.com (O.K.); nino.mimica@gmail.com (N.M.); 3 School of Medicine, University of Zagreb, 10000 Zagreb, Croatia; 4 Faculty of Education and Rehabilitation Sciences, University of Zagreb, 10000 Zagreb, Croatia; 5 Glycobiology Laboratory, Genos Ltd., 10000 Zagreb, Croatia; glauc@genos.hr; 6 University of Applied Sciences Hrvatsko Zagorje Krapina, 49000 Krapina, Croatia

**Keywords:** post-traumatic stress disorder, fucosyltransferase 8 (FUT8), *FUT8*-related polymorphisms, glycosylation, haplotype, N-glycans, plasma

## Abstract

The molecular underpinnings of post-traumatic stress disorder (PTSD) are still unclear due to the complex interactions of genetic, psychological, and environmental factors. Glycosylation is a common post-translational modification of proteins, and different pathophysiological states, such as inflammation, autoimmune diseases, and mental disorders including PTSD, show altered N-glycome. Fucosyltransferase 8 (FUT8) is the enzyme that catalyzes the addition of core fucose on glycoproteins, and mutations in the *FUT8* gene are associated with defects in glycosylation and functional abnormalities. This is the first study that investigated the associations of plasma N-glycan levels with *FUT8*-related rs6573604, rs11621121, rs10483776, and rs4073416 polymorphisms and their haplotypes in 541 PTSD patients and control participants. The results demonstrated that the rs6573604 T allele was more frequent in the PTSD than in the control participants. Significant associations of plasma N-glycan levels with PTSD and *FUT8*-related polymorphisms were observed. We also detected associations of rs11621121 and rs10483776 polymorphisms and their haplotypes with plasma levels of specific N-glycan species in both the control and PTSD groups. In carriers of different rs6573604 and rs4073416 genotypes and alleles, differences in plasma N-glycan levels were only found in the control group. These molecular findings suggest a possible regulatory role of *FUT8*-related polymorphisms in glycosylation, the alternations of which could partially explain the development and clinical manifestation of PTSD.

## 1. Introduction

Post-traumatic stress disorder (PTSD) is a severe trauma- and stress-related disorder with characteristic symptoms that span across different emotional, cognitive, and psychological domains [1,2]. It is often accompanied with severe mental and somatic comorbidities such as depression, alcohol and substance abuse, suicidal behavior, and cardiovascular and metabolic diseases, leading to a higher probability of adverse health outcomes and shorter life expectancy among affected individuals [1,2]. The heterogeneity of PTSD symptoms, a broad spectrum of affected molecular systems and circuits, and complex molecular interactions between the inherited and acquired factors that contribute to the risk and progression of PTSD represent the confounding elements in the identification and validation of PTSD biomarkers.

Recent studies demonstrated the involvement of altered N-glycosylation in several psychiatric disorders [3,4,5,6], including PTSD [7,8], as well as in various somatic pathological and inflammatory states, such as cardiovascular, metabolic and pulmonary diseases, infection, autoimmune disorders, and cancer [9,10]. N-glycosylation is the most common co- and post-translational modification of proteins in the eukaryotic cells that involves the addition of sugar moieties, with N-acetylglucosamine (GlcNAc), N-acetylgalactosamine (GalNAc), galactose, mannose on a consensus asparagine-containing sequence, sialic acid and fucose representing the most frequent added sugars [11]. The diversity of sugar residues and their possible combinations and linkages affect the physio-chemical properties of the glycoproteins on a molecular level, which can result in their altered biological function [12]. For instance, the galactosylation of the immunoglobulin G (IgG)-attached N-glycans acts as a modulator of its inflammatory activity by affecting the complement-dependent cytotoxicity [9,13]. Moreover the α2,6-sialylation of the IgG is associated with an anti-inflammatory response [14,15], while a terminal hypersialylation in the tumor cells can affect leukocyte migration, metastasis, and tumor progression [16].

Fucosylation is a molecular process in which fucose from the donor molecule guanosine biphosphate fucose (GDP-Fuc) is added to the acceptor molecules, such as terminal galactose via the α1,2 bond or the subterminal and innermost GlcNAc via the α1,3/4 and the α-1,6 glycosidic bond, respectively [17]. While there are several fucosyltransferases (FUT3-7, FUT9-11) that catalyze the addition of fucose via the α1,3/4 linkage, resulting in the antennary fucosylated glycoproteins, fucosyltransferase 8 (FUT8) is the only enzyme in mammals with α1,6 fucosyltransferase activity, resulting in a formation of the core-fucosylated N-glycans [17].

Fucose-containing glycans are involved in blood antigen synthesis and transfusion reactions, leukocyte–endothelial adhesion mediated by selectin, and host–microbe interactions [18,19,20,21]. In addition, the core-fucosylated glycans, predominantly attached to the IgG, are strongly linked to metastasis [22], possibly by affecting antibody-dependent cellular cytotoxicity (ADCC) [23], programmed cell death protein 1 (PD-1) [24], and transforming growth factor β1 (TFG-β) receptor [9,25].

Several pathological states such as autoimmune disorders, cardiovascular diseases, and cancer, and neuropsychiatric disorders including PTSD, have been associated with altered fucosylation molecular patterns in humans, where the core-fucosylation is crucial in maintaining the homeostasis of the organism [9,20,21]. Bi-allelic mutations in the *FUT8* gene, resulting in defective FUT8 α1,6 fucosyltransferase activity and the absence of the core-fucosylated N-glycans, lead to the development of the severe metabolic congenital disorder of glycosylation with defective fucosylation 1 (FUT8-CDG) in humans [26]. Moreover, in mice, the complete deletion of this gene is highly lethal and causes severe growth retardation, emphysema-like changes in the lungs, and schizophrenia-like symptoms [27], possibly by interfering with TGF-1 receptor activation, vascular endothelial cell growth factor receptor-2 (VEGF-2) expression, EGF receptor signaling, and integrin α3β1-mediated cell adhesion [26,28,29].

Genetic influence on glycosylation and specifically core-fucosylation is still not completely understood. Unlike protein synthesis, glycosylation is a non-template-driven molecular process regulated by various microenvironmental and intracellular changes [30]. However, glycoenzymes and other proteins included in glycan formation and modification are encoded in a genome and their availability, expression, and activity are regulated at the transcriptional, translational, or post-translational levels [30,31]. It is estimated that approximately 1% of a genome encodes for glycoenzymes, although large variations in heritability were observed depending on the N-glycan structures [30,32]. Estimated IgG N-glycans heritability of >50% and total plasma N-glycan heritability ranging from 17–74% (average 60%) [32,33] have been reported.

Studies of the first plasma glycome, GWAS [34], and the following replication, GWA [35,36], identified the association of several loci with the levels of plasma N-glycans, of which most were located in the *FUT8*, *FUT6*, and *HNF1A* gene regions. HNF1A is considered a major molecular regulator of fucosylation, possibly by regulating the expression of FUT8 and antennary fucosyltransferases (FUT3, FUT5, FUT6). The genetic and epigenetic associations of the *HNF1A* gene with levels of several highly branched and sialylated plasma N-glycans, as well as with the core- and antennary-fucosylated IgG N-glycans, were reported in recent studies [37,38]. Numerous single nucleotide polymorphisms (SNPs) were suggested to significantly affect the IgG and plasma N-glycan composition, among which the most prominent ones were located within or near the *FUT8* locus and associated with the A2 and A2BG2 glycan levels, as well as with the core-fucosylated FA2G2 and FA3B1G1 N-glycans [34,35,36].

Therefore, this study aimed to investigate the possible association of the plasma N-glycan levels in patients with PTSD and in control participants, with four polymorphisms related to the *FUT8* gene region (rs6573604, rs11621121, rs10483776, and rs4073416), which have shown a high genome-wide significance in glycosylation during previous GWA studies.

## 2. Results

Genotype and allele frequencies of the rs6573604, rs11621121, rs10483776, and rs4073416 polymorphisms located in the *FUT8* gene region were determined in a total sample of 541 participants. Minor allele frequencies (MAF) and corresponding Hardy–Weinberg equilibrium (HWE) were determined for each polymorphism and are represented in Table 1. The MAFs for rs6573604, rs11621121, rs10483776, and rs4073416 polymorphisms were 19.0%, 44.0%, 20.7%, and 41.6%, respectively, in accordance with the estimated MAFs in the European population, as reported in the Allele Frequency Aggregator (ALFA) database [39]. The genotype distributions of the rs11621121, rs10483776, and rs4073416 polymorphisms were in the expected HWE, while the distribution of the rs6573604 genotypes deviated from the HWE (Table 1).

**Figure 1:**
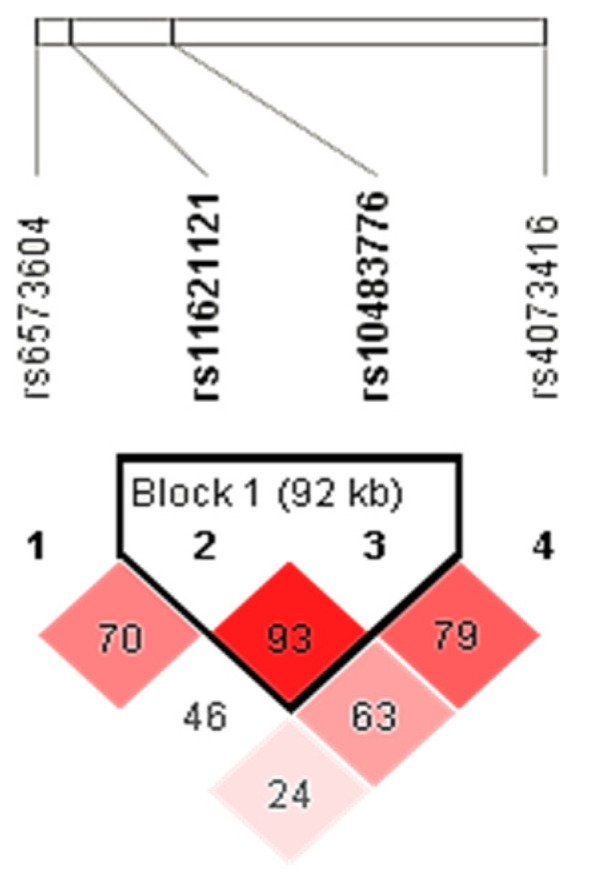
LD plot of the rs6573604, rs11621121, rs10483776, and rs4073416 polymorphisms located in the *FUT8* gene-related region. Pairwise LD value (×100) for the rs11621121 and rs10483776 combination, as denoted in a bright red rectangle (D′ = 93), indicates a strong link between these two polymorphisms.

Haplotype analysis showed a weak linkage disequilibrium (LD) between all four tested polymorphisms (D′ × 100 = 24); however, the rs11621121 and rs10483776 polymorphisms were in a strong LD (D′ × 100 = 93) (Figure 1). Therefore, the haplotypes for the rs11621121 and rs10483776 polymorphism block were determined for each subject using an expectation–maximization algorithm. The most common was the TA haplotype, which was represented in more than half of the subjects (55.5%), followed by the CA (25.9%) and the CG (21.6%) haplotypes. The rarest was the TG haplotype (0.5%), which was excluded from further analysis due to its low frequency (<1%).

### 2.1. Association of the FUT8-Related Polymorphisms with PTSD

Differences in the distribution of the genotypes, alleles, and haplotypes of the rs6573604, rs11621121, rs10483776, and rs4073416 polymorphisms between the control participants and the patients with PTSD were determined using a χ^2^-test. There were no significant differences in the observed frequencies of the rs11621121, rs10483776, and rs4073416 genotypes and alleles (Table 2), nor the rs11621121–rs10483776 haplotypes between these two groups of participants (Table 3). However, the C allele of the rs6573604 polymorphism was more frequently present in the control participants (*p* = 0.017; R = 1.6) compared to the patients with PTSD, who had a higher prevalence of the T allele than the control participants (Table 2).

### 2.2. Differences in the N-Glycome between the PTSD and the Control Group

Multiple linear regression was used to determine the effect of age, body mass index (BMI), and diagnosis on the levels of different plasma N-glycan species. The significant effect of age on the N-glycome has been previously recognized [7,33,40,41] and it was confirmed in our model as well. Specifically, age was a main predictor for the plasma levels of most N-glycans, as reported previously (full data available on request), while BMI did not contribute significantly to the levels of plasma N-glycans [8]. Therefore, the residuals obtained from fitting the linear model of each glycan peak depending on the age were used in a further statistical analysis to correct this effect [7].

**Figure 2:**
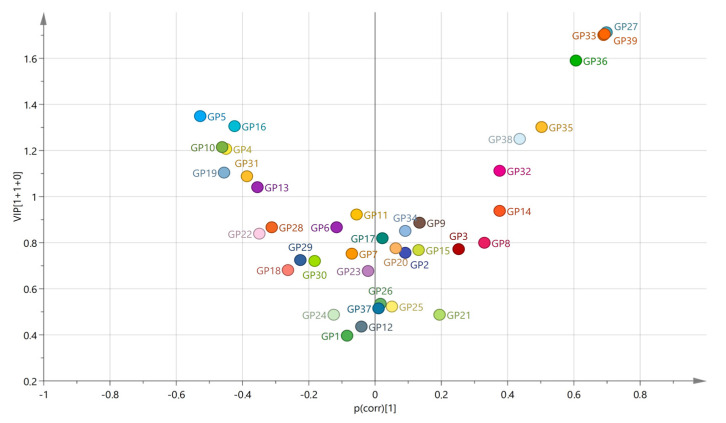
Volcano plot plotting the variable importance in the projection (VIP), acquired in the OPLS-DA model, against the correlation coefficient values (p(corr)) for all included variables between the PTSD patients and the control participants. Colored according to the identifiers.

As diagnosis was also a significant predictor of the several N-glycan levels in the plasma, we performed the Supervised Orthogonal Partial Least Square–Discriminant Analysis (OPLS-DA), with all age-corrected levels of plasma N-glycans listed as variables. OPLS-DA acquired variable importance in the projection (VIP) scores, and correlation coefficients values—p(corr) showed the intermediate correlation for several N-glycan peaks in discriminating between the control and PTSD groups (Figure 2, Supplementary Figure S1, Supplementary Table S2). Among the strongest associated N-glycans in this model were also those that we previously reported as being significantly altered between the patients with PTSD and the control participants [8]. Therefore, in this study, we have focused mainly on the association of the polymorphisms in the *FUT8*-associated region with the relative levels of the N-glycans in the plasma, but due to differences in the N-glycome between the enrolled diagnostic groups, we performed the analysis separately for the patients with PTSD and for the control participants.

### 2.3. Association of the FUT8-Related Polymorphisms with the N-Glycan Levels

The level of each N-glycan peak was analyzed in the carriers of different genotypes (genetic model) and alleles (allelic model) of the rs6573604, rs11621121, rs10483776, and rs4073416 polymorphisms, as well as the rs11621121–rs10483776 haplotypes, separately for the control and the PTSD group. The significance level was corrected for the number of analyzed peaks using the False Discovery Rate (FDR) method (Benjamini–Hochberg). The N-glycan moieties whose levels differed significantly between the carriers of different genotypes or alleles are reported in Table 4 for the control group and in Table 5 for the PTSD group. The statistical data for all analyzed N-glycan peaks are available in Supplementary Tables S3–S5. The strongest link of the plasma N-glycan levels was observed with the rs11621121 and rs10483776 polymorphisms, and their haplotype block in both diagnostic groups, while the significant associations of the rs6573604 and rs4073416 polymorphisms with the plasma N-glycan levels were found only in the control group.

**Figure 3:**
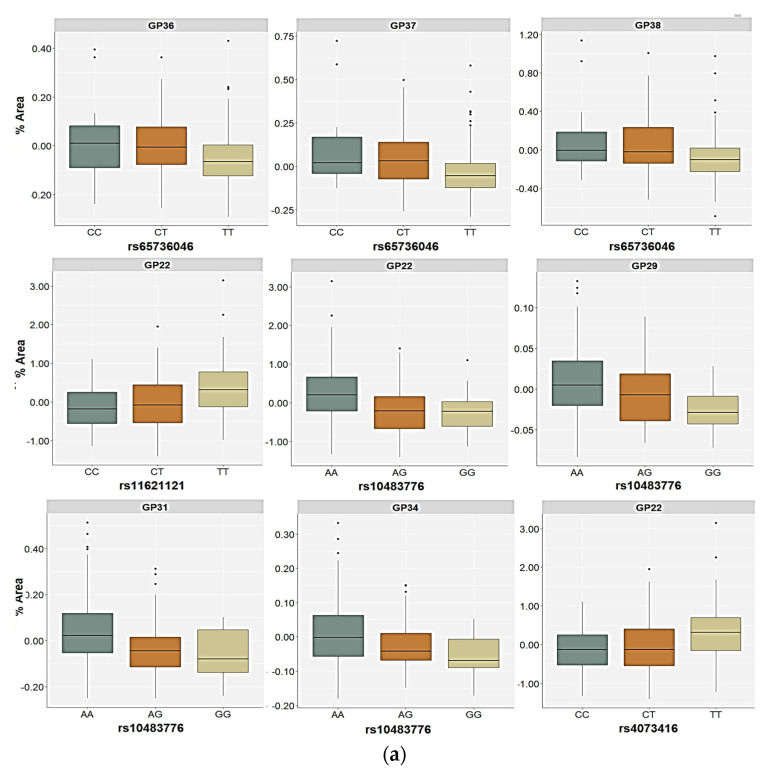

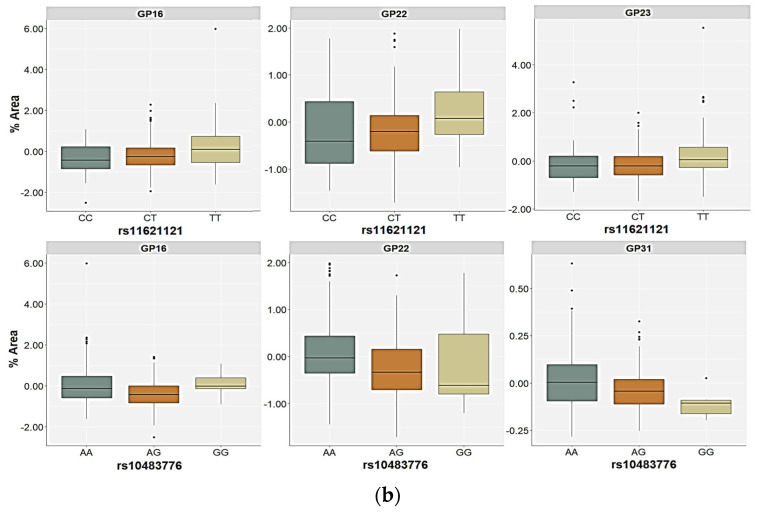
Relative distribution of the plasma N-glycan levels in the carriers of different genotypes for the rs6573604, rs11621121, rs10483776, and rs4073416 polymorphisms in the (**a**) control and (**b**) PTSD group. The central box represents the interquartile range of the age-adjusted percentage of the total glycan peak area, the middle line represents the median, the vertical line extends from the minimum to the maximum value, and separate dots represent the outliers.

Polymorphism rs6573604 was associated with the levels of the GP36 (*p* = 0.020), GP37 (*p* = 0.013), and GP38 (*p* = 0.007) glycans in the plasma of the healthy control subjects (Table 4, Supplementary Table S3). These three glycan peaks share the same tetra-antennary, galactosylated, and sialylated N-glycan structure (A4G4S4), but they differ in the type of linkage (α2,3 or α2,6-bond) by which sialic acid is attached to galactose (Supplementary Table S1). For the rs6573604 polymorphism, the TT homozygotes had the lowest plasma levels of the GP36, GP37, and GP38 glycans compared to the CT (*p* < 0.001, post hoc Dunn test) and the CC carriers (*p* < 0.016, post hoc Dunn test) (Figure 3). The association of the T allele with a lower abundance of these N-glycans was confirmed in the allelic model (*p* = 0.003 for GP36, *p* = 0.001 for GP37 and GP38) (Table 4, Supplementary Table S3). Although several N-glycan peaks showed a nominal association with the rs6573604 polymorphism in the patients with PTSD, of which the GP29 glycan was the most prominent, none of the N-glycan peaks reached significance after the correction for multiple testing (Supplementary Table S3).

**Figure 4:**
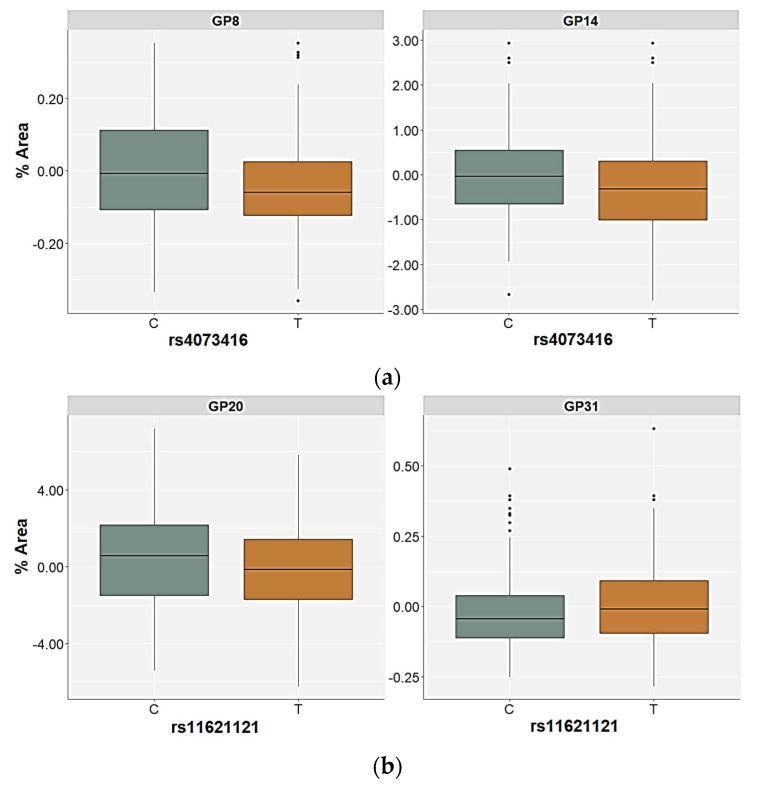
Relative distribution of the plasma N-glycan levels in the carriers of different alleles for (**a**) the rs4073416 polymorphism in the control group and (**b**) the rs11621121 polymorphism in the PTSD group. The central box represents the interquartile range of the age-adjusted percentage of the total glycan peak area, the middle line represents the median, the vertical line extends from the minimum to the maximum value, and separate dots represent the outliers.

For the rs11621121 polymorphism, the TT homozygotes (and the T allele carriers) had significantly higher levels of the GP22 (FA2G2S2) glycan in plasma compared to the CC and the CT carriers (*p* < 0.001, post hoc Dunn test) or C allele carriers (*p* = 0.001), both in the control as well as in the PTSD group (Table 4 and Table 5, Figure 3). There were no other significant differences in the plasma N-glycan levels associated with this SNP in the control participants. However, the GP08 (A2G2) glycan levels were higher in the carriers of the CC genotype or the C allele, but only at a nominal significance level (Supplementary Table S3). The relative distribution of the GP16 (FA2G2S1), GP20 (A2G2S2), GP23 (FA2BG2S2), and GP31 (FA3G3S3) glycan levels differed significantly between the carriers of different rs11621121 genotypes or alleles in the PTSD group (Table 5, Supplementary Table S3). Specifically, the TT homozygotes and the T allele carriers had higher plasma levels of the GP16 and GP23 glycans compared to the PTSD patients carrying the CC and CT genotypes (post hoc Dunn *p* = 0.004 for GP16; *p* = 0.003 for GP23) or the C allele (*p* = 0.020 for GP16; *p* = 0.013 for GP23, Figure 3). A significant association of the GP20 and GP31 glycan levels in the plasma with the rs11621121 polymorphism was observed only in the allelic model, in which a lower abundance of the GP20 glycan levels (*p* = 0.039) and higher abundance of the GP31 glycan levels (*p* = 0.045) in the plasma were detected in the T allele carriers (Figure 4).

In both the PTSD and control subjects, the rs10483776 polymorphism was associated with the GP22 (FA2G2S2) and GP31 (FA3G3S3) glycan levels in plasma (Table 4 and Table 5). The GP22 levels were significantly higher in the AA genotype carriers compared to the AG (*p* < 0.001, post hoc Dunn test) and the GA carriers (*p* = 0.041, post hoc Dunn test) in the control group, as well as higher in comparison to the PTSD patients who were heterozygous for this polymorphism (*p* = 0.010, post hoc Dunn test) (Figure 2). Similarly, the GP31 glycan plasma levels were the highest in the AA homozygotes and the lowest in the carriers of the rs10483776 GG genotype (Figure 3). Additional associations of the rs10483776 SNP were observed with the plasma GP29 (FA3G3S3) and GP34 (A4G4S3) glycan levels in the control participants in both the genetic and allelic model. Specifically, the AA homozygotes had higher levels of these N-glycans compared to heterozygotes (*p* = 0.011 for GP29; *p* < 0.001 for GP31, post hoc Dunn test) and GG genotype carriers (*p* = 0.004 for GP29; *p* = 0.011 for GP31, post hoc Dunn test). The GP16 (FA2G2S1) glycan levels in the plasma of PTSD patients were also significantly different between the carriers of different genotypes, but not alleles, where the rs10483776 heterozygotes had the lowest levels of the GP16 glycan compared to the AA (*p* < 0.001, post hoc Dunn test) and GG homozygotes (*p* = 0.041, post hoc Dunn test) (Table 4 and Table 5, Figure 3).

The rs4073416 polymorphism was associated with the plasma GP08 (A2G2), GP14 (A2G2S1), and GP22 (A2G2S2) glycan levels in the control participants (Table 4). The TT homozygotes had higher levels of GP22 glycan levels in the plasma compared to the CC homozygotes and the CT carriers (post hoc Dunn *p* = 0.001). The association of the T allele with the higher plasma levels of the GP22 glycan was also confirmed in the allelic model (*p* = 0.016) (Table 4). In contrast, the association of the rs4073416 polymorphism with the levels of the GP08 and GP14 glycans in the plasma was only observed in the allelic model, in which the T allele carriers had lower plasma levels of these glycans, representing the non-fucosylated, biantennary N-glycans with lower sialylation levels (Figure 4).

**Figure 5:**
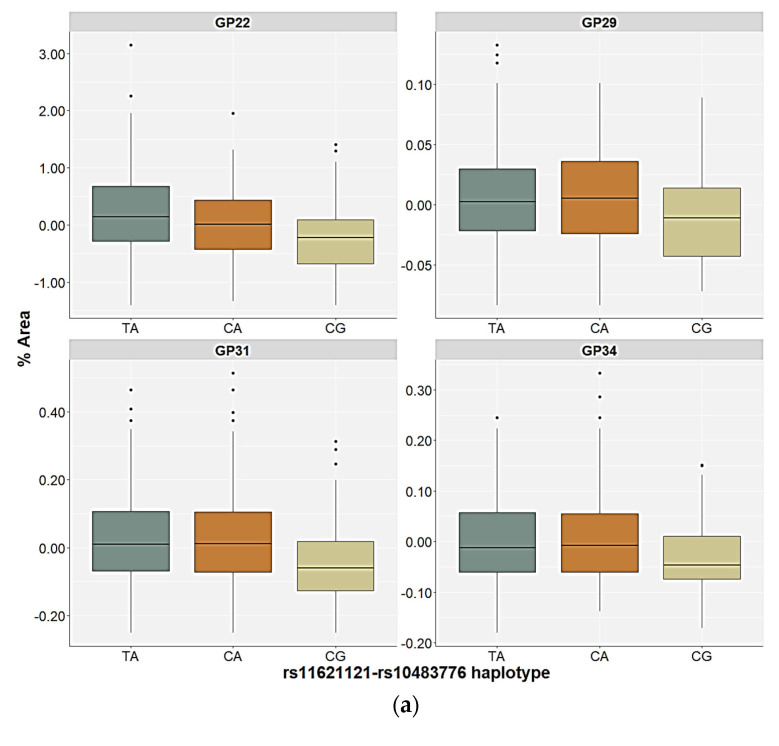

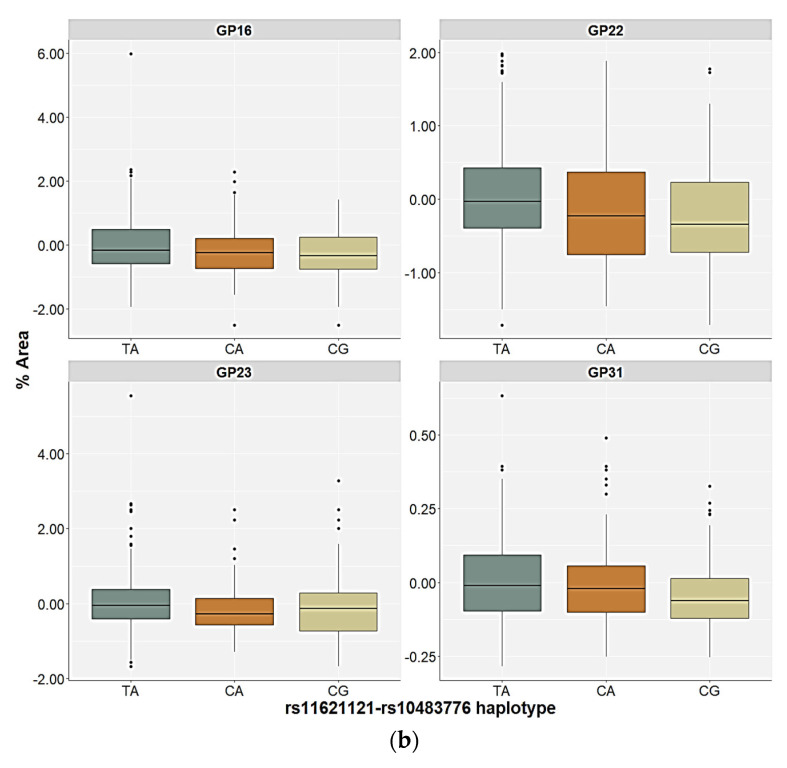
Relative distribution of the plasma N-glycan levels in the carriers of the different rs11621121–rs10483776 haplotypes in the (**a**) control and (**b**) PTSD group. The central box represents the interquartile range of the age-adjusted percentage of the total glycan peak area, the middle line represents the median, the vertical line extends from the minimum to the maximum value, and separate dots represent the outliers.

The control participants and the PTSD patients carrying the different rs11621121–rs10483776 haplotypes showed differences in the relative distribution of the GP22 and GP31 glycan levels in the plasma. However, the differences in the plasma GP29 and GP34 glycan levels were only observed in the control participants, and the GP16 and GP23 glycan levels in the plasma only differed in the patients with PTSD (Table 4 and Table 5). All four significant N-glycan peaks followed the same distribution pattern across the carriers of different haplotypes in the control group. The lowest plasma levels of the GP22, GP29, GP31, and GP34 glycan levels were associated with the CG haplotype compared to the TA (*p* < 0.001, post hoc Dunn test) and the CA haplotype carriers (*p* < 0.010, post hoc Dunn test), who did not differ significantly in the relative distribution of these N-glycan levels (Figure 5). In the PTSD group, the CG haplotype and the CA haplotype carriers had lower levels of the GP16, GP22, and GP23 glycan levels in the plasma compared to the TA haplotype carriers (*p* < 0.001). Moreover, the lowest plasma levels of the GP31 glycans were associated with the CG haplotype compared to the TA (*p* < 0.001) and the CA (*p* = 0.009) haplotypes of the PTSD patients (Figure 5).

## 3. Discussion

This study is the first association study to analyze the molecular link between plasma N-glycan levels, different genetic polymorphisms located in the *FUT8*-linked region, and PTSD. Our study found a significant association of the plasma N-glycan levels with PTSD, as well as with the rs6573604, rs11621121, rs10483776, and rs4073416 polymorphisms. We detected the strongest association of the rs11621121 and rs10483776 polymorphisms, as well as their haplotype block with the plasma levels of the core-fucosylated bi- and tri-antennary N-glycan species in both the control and the PTSD groups. On the other hand, for the rs6573604 and rs4073416 polymorphisms, the differences in plasma N-glycan levels among the carriers of the different genotypes and alleles were only found in the control group. Moreover, a possible association between the T allele of the rs6573604 polymorphism and PTSD was detected, because this allele was more frequent in the patients with PTSD than in the control participants, who were more frequent carriers of the C allele.

Significant differences in N-glycome between the patients with PTSD and the control participants, as we have reported previously [8], were reflected mainly in the elevated plasma levels of the tri- and tetra-antennary, highly galactosylated, and sialylated N-glycan structures and in the decreased plasma levels of the core-fucosylated biantennary N-glycans with the lower degree of sialylation, which are mainly derived from the IgG [42]. The presence of the core-fucose acts as a “safety switch” against the ADCC by significantly decreasing the affinity of the IgG to FcγRIIIA and FcγRIIIB receptors and exerting its anti-inflammatory effect [7,9,43,44,45]. The lower degree of the core-fucosylation in the IgG N-glycans, as well as the increased complexity and sialylation of the plasma N-glycans, which are seen in PTSD, are also observed in inflammation, autoimmune diseases, cancer [10,46,47], and other psychiatric disorders, such as schizophrenia [4] and major depressive disorder [5] in several human studies.

To the best of our knowledge, there are no reported data yet on the *FUT8*-related polymorphisms associated with PTSD, while molecular relationships with other psychiatric disorders and neurodegenerative diseases are based on GWAS reports. The GWA studies linked several variants located in a close proximity or within the *FUT8* gene region with schizophrenia [48,49], major depressive episodes [50], cognitive decline in Alzheimer’s disease [51], and multiple sclerosis [52], although any replication of these results is missing. However, *FUT8* polymorphisms have been associated with the levels of high-density lipoprotein cholesterol (rs10483776) [53,54], hypertension [55], and chronic obstructive pulmonary disease [56,57], which are common comorbidities in PTSD [1].

In our study, the T allele of the rs6573604 polymorphism was associated with higher risk of PTSD, as well as with the lower plasma levels of tetra-antennary, tetra-galactosylated, and tetra-sialylated (A4G4S4) N-glycans in the control participants. Previous reports on the association of the rs6573604 polymorphism with N-glycan levels demonstrated a positive effect of the G allele on the A2 N-glycan levels in the male population, but this effect was not related to the levels of highly-branched N-glycans [35]. This is not surprising, since this type of N-glycan moieties usually has low heritability scores [32,33], and its almost exclusive source in plasma is acute-phase alpha-1-acid glycoprotein (AAG) [42], whose concentration in the serum rises considerably in response to inflammatory stimuli [58]. Highly sialylated tri- and tetra-antennary N-glycans, which exhibit the immunomodulatory function by regulating the complement system and the transport of lipophilic molecules [42,59], are the predominant N-glycans on AAG, and a detected increase in the levels of these N-glycans in plasma often reflects ongoing inflammation [60,61]. Although genetic influence on these processes is considered negligible, low heritability N-glycans are often influenced by epigenetic factors, such as changes in DNA methylation [62]. In particular, recent studies have shown a significant correlation between the methylation of the *HNF1A* gene and the abundance of the highly sialylated tri- and tetra-antennary N-glycans including the A4G4S4 glycan [37,38]. Since the rs6573604 polymorphism is located within the microRNA 4708 gene (*MIR4708*), in close proximity of the 5′ end of the *FUT8* gene, it is possible that it affects the plasma levels of these N-glycans through molecular epigenetic mechanisms [63].

Additionally, we found several other associations between the rs11621121 and rs10483776 polymorphisms located in the regulatory and intron regions of the *FUT8* gene, respectively, and plasma levels of N-glycans containing mostly the core-fucosylated, bi- and tri-antennary, galactosylated, and sialylated structures. The strongest association in both diagnostic groups was detected with plasma GP22 (FA2G2S2) and GP31 (FA3G3S3) glycan levels, which were increased in the individuals carrying the T allele of the rs11621121 polymorphism, the A allele of the rs10483776 polymorphism, or the TA rs11621121–rs10483776 haplotype in both the control and PTSD individuals. Other associations of the N-glycan levels in the plasma with the rs11621121 polymorphism were mostly noticeable in the PTSD group, where the carriers of the T allele had increased plasma levels of GP16 (FA2G2S1) and GP23 (FA2BG2S2) glycans, and lower plasma levels of the non-fucosylated GP20 (A2G2S2) glycan. In contrast, the effect of the rs10483776 polymorphism (the A allele) on the plasma N-glycan levels was only observed in the control group, and was associated with higher plasma concentrations of GP29 (FA3G3S3) and GP34 (A4G4S3) glycans. As for GP16 glycan, the effective allele of the rs10483776 polymorphism could not be distinguished, as the heterozygotes had the lowest plasma levels of this N-glycan in the PTSD group. These results are in agreement with previous GWAS that reported the negative effect of the rs10483776 G allele on the plasma levels of the N-glycans DG6, DG10, GP10, and C-FUC, which represent the core-fucosylated, di-galactosylated and sialylated N-glycans with two or three antennae [34,35,36]. For the rs11621121 polymorphism, the observed significant positive association of the C allele with the plasma levels of non-fucosylated A2G2S2 glycan, as well as its nominal association with the A2G2 glycan levels in the plasma, is consistent with the previous studies demonstrating a positive effect of the rs11621121 G allele on the levels of the bi-antennary N-glycans without core-fucose [34,35,36].

Furthermore, our study showed a significant association of the C allele of the rs4073416 polymorphism with lower plasma levels of GP22 glycan and higher plasma levels of GP08 (A2G2) and GP14 (A2G2S1) glycans, both representing the afucosylated, bi-antennary, and di-galactosylated N-glycans with lower sialylation levels, although these results were limited to the control group. Previous GWAS reported a positive association of the G allele with bi-antennary agalactosylated N-glycans (A2); however, this effect was only observed in women [33]. These inconsistent results could be explained by the already-known gender differences in the N-glycome [33], as only male subjects participated in our study.

The high heritability of the core-fucosylated N-glycans, GP16 (FA2G2S1), GP22 (FA2G2S2), GP23 (FA2BG2S2), and GP31 (FA3G3S3), which were significantly associated with the *FUT8* polymorphisms in our study, has been reported previously [32]. In contrast, the non-fucosylated GP20 (A2G2S2) and GP14 (A2G2S1) glycans, which are the most abundant N-glycans present in several plasma glycoproteins, exhibit low heritability scores [32]. This could be due to the fact that most of the core-fucosylated, bi- and tri-antennary structures show greater protein specificity than the non-fucosylated N-glycans, with the exception of GP08 (A2G2) glycan, which is only present in apolipoprotein B-100 [42]. The plasma levels of the FA2G2S1 and the FA2BG2S2 glycans derive almost entirely from the IgM and the IgG-A22 glycan, respectively [64]. Although the FA2G2S2 glycan is mainly found in immunoglobulins, it is not exclusively restricted to them, but may also be present in other plasma proteins involved in the immunological and antioxidant response, such as alpha-1-glycoprotein, haptoglobin, serotransferrin, and to a lesser extent in other proteins [42]. The primary source of FA3G3S3 glycan is vitronectin [42], a glycoprotein involved in cell adhesion, extracellular matrix binding, and blood coagulation [65], and its glycosylation pattern mainly includes non-fucosylated N-glycans, with the site-specific core-fucosylation being a possible indicator of malignant changes, such as hepatocellular carcinoma [66].

In the patients with PTSD, almost all significant associations of the tested polymorphisms were observed with the plasma levels of N-glycans that were core-fucosylated, whereas in the control group, we found an additional association of all tested polymorphisms with the plasma levels of several non-fucosylated N-glycans linked to the acute-phase proteins detected in various inflammatory states. In a recent pilot study that evaluated patients with PTSD due to civilian trauma using in vivo neuro 2D MR spectroscopy, an increase in two fucose-α(1–2)-glycans and the appearance of the substrate α-fucose in the brain was detected [67]. Findings from animal models have already shown that fucose-α(1–2)-glycans, observed at the synapse of the neurons, play a role in several neurological processes such as neuronal development and learning [68,69]. This may indicate the role of fucose in neuronal communication and signal transduction, which can contribute to the altered neurobiology and pathogenesis of PTSD [67]. In contrast, the hypersialylation of plasma proteins could contribute to increased inflammation. Specifically, it could modulate the platelet activation through the interaction of sialic acid with P- and E-selectins [5], or by protecting the acute-phase proteins from protease digestion and therefore maintaining their abundance in the bloodstream [4]. It is possible that the underlying chronic inflammation, which is considered a hallmark of PTSD symptomatology [70,71], conceals the potential genetic influence on the plasma levels of some of the investigated N-glycans, with different microenvironmental and epigenetic factors potentially contributing more extensively to their abundance and release in a non-homeostatic state, such as PTSD.

As mentioned previously, altered glycosylation plays an important role in modulating the immune response mainly through lectins (galectins, selectins, siglecs, etc.), the carbohydrate-binding proteins that can be found free or expressed on the cell surface of many immune cells, such as NK and T-cells, B-cells, dendritic cells, and leukocytes, as well as in endothelial cells and platelets [72]. The complex interaction of glycan-containing motifs on different cell receptors is involved in microbial recognition and elimination, cell adhesion, antigen-specific immune response, tumor cell identification, and modulation of the immune cell function [10]. These findings have enabled the development of potential strategies for the treatment of different autoimmune diseases and carcinomas. For example, the gp120 glycoprotein expressed on the surface of the Human Immunodeficiency Virus 1 (HIV-1) envelope enables the virus to evade detection by the host immune system. Differential glycosylation of the gp120 glycoprotein can be used not only for the identification of different types of HIV-1 clades, but also for the prediction of vaccine treatment efficacy and the production of more specific and optimized vaccination regimens [73,74]. Moreover, the glycoengineering of antibodies and intravenous Ig with elevated galactosylation and α2,6-linked sialylation, which exhibits anti-inflammatory properties, could be used in the treatment of chronic diseases such as rheumatoid arthritis (RA), systemic lupus erythematosus (SLE), and inflammatory bowel disease (IBD), as this type of IgG glycoform is often decreased in the aforementioned diseases [10,13,15,75]. On the other hand, afucosylated monoclonal antibodies could be applied to treat certain types of cancer due to enhanced ADCC [76]. In a recent pilot study, differences in baseline plasma glycosylation patterns were found in female MDD patients depending on the efficacy of antidepressant treatment [5]. Since PTSD and depression share similar glycosylation patterns and therapeutic strategies, and depression is one of the major comorbidities in PTSD [1], this finding may have the potential to estimate the treatment response in PTSD patients. However, it is important to investigate the possible role of gender and levels of sex hormones on this effect, as they can affect the N-glycan concentrations and neurotransmitter metabolism [33,77].

High-resolution separation techniques, strict exclusion criteria, and adjustments for the effect of the age and multiple testing contribute to the strength of the study. Moreover, the inclusion of solely male, Caucasian participants of similar age in both diagnostic groups, the additional control for the effects of age, BMI, and smoking, and the exclusive focus on combat-related PTSD reduce the possible effect of these confounding variables on N-glycan levels and support the findings of this study. However, the relatively small sample size for a genetic study, biological parameters that may have been overlooked in this study, and still-unresolved molecular mechanisms by which plasma N-glycans influence the signaling pathways pose challenges to the unequivocal interpretation of the obtained results. Additional multidisciplinary experiments in animal models of PTSD, such as immunohistochemical and Western blot analysis for the determination of FUT8 expression in different brain areas, high-performance liquid chromatography (HPLC) for neurotransmitter studies, and positron emission tomography (PET) scans and functional magnetic resonance imaging (fMRI) of patients with PTSD, would strengthen the current findings. In addition, the interaction of these SNPs with the expression levels of the *FUT8* gene and plasma N-glycome and the validation of our results in a larger number of participants, as well as in women, in individuals of different ethnicities, and in individuals exposed to different types of trauma, could provide more insight and overcome the limitations of this study.

Nonetheless, this is the first study to report the molecular associations of *FUT8*-related genetic polymorphisms with the levels of the plasma N-glycans in a relatively homogeneous group of PTSD patients and control participants. The differentiation between the non-fucosylated and the core-fucosylated N-glycan levels by different alleles or haplotypes of the *FUT8*-related polymorphisms is consistent with the known biological role of FUT8, and it adds supporting evidence for the genetic effects on the core-fucosylation. Moreover, the variations in the plasma N-glycome could reflect the changes in the protein composition in the plasma, thus providing a better insight into the immunological and pathological state of the organism at the molecular level, using this relatively easily obtainable source of biomarkers, and revealing novel and personalized strategies for the treatment of PTSD.

## 4. Materials and Methods

### 4.1. Participants

This study enrolled a total of 541 unrelated, Caucasian, male participants, of whom 295 were war veterans with PTSD with a median age of 55 (51; 61), and 246 were healthy control participants not exposed to war trauma within the same age range (median age of 55 (48; 62)). Participants were recruited at the University Psychiatric Hospital Vrapce, Zagreb, and diagnosed with current and chronic PTSD using the Structured Clinical Interview (SCID) based on the DSM-5 criteria [78] and the Clinician Administered PTSD Scale (CAPS) [79]. The majority of participants with PTSD had moderately severe PTSD symptoms (median CAPS scores of 86 (78; 88)), with a similar number and type of trauma (combat-related). All participants were evaluated using the same diagnostic instruments according to the DSM-5 criteria and the International Classification of Diseases (ICD-10) to exclude the possible presence of other psychiatric disorders, such as schizophrenia, bipolar disorder, adult attention deficit hyperactivity disorder (ADHD), substance and alcohol abuse, Alzheimer’s disease, somatic diseases leading to altered liver function, and current use of antihypertensive, antidiabetic, and lipid-lowering medications. The patients with PTSD did not receive psychopharmacological therapy in the 30 days before the blood collection. The study was approved by the Ethics Committee of the University Psychiatric Hospital Vrapce, Zagreb, and the Bioethics Committee of the Rudjer Boskovic Institute, Zagreb, Croatia. All participating subjects signed an informed consent form prior to the blood sampling, in accordance with the Helsinki Declaration (1975), revised in 2013.

### 4.2. Blood Processing

The blood samples were collected in the morning using BD Vacutainer™ glass collection tubes (Becton, Dickinson and Company, Franklin Lakes, NY, USA) with acid citrate dextrose (ACD) anticoagulant, and were processed on the same day. Platelet-poor plasma used for the glycomic analysis was isolated using the series of centrifugation (3 min at 1811× *g*, followed by 15 min at 5031× *g*), as described previously [80], while DNA from the peripheral blood was isolated using a salting out method [81]. The plasma samples were immediately frozen and stored at −80 °C and DNA samples were stored at +4 °C until further analysis.

### 4.3. Determination of N-Glycan Levels in the Plasma

The relative distribution of the N-glycan levels derived from the total plasma glycoproteins was determined using hydrophilic interaction high-performance liquid chromatography (HILIC), as described previously [82]. Briefly, the protein denaturation from the platelet-poor plasma was performed with 2% (*w*/*v*) sodium dodecyl sulfate (SDS) (Invitrogen, Camarillo, CA, USA) for 10 min at 65 °C, followed by the addition of 4% (*v*/*v*) Igepal CA630 (Sigma Aldrich, St. Louis, MO, USA). N-glycan release from the proteins was accomplished by adding 1.2 U of the PNGase F (Promega, San Luis Obispo, CA, USA), followed by overnight incubation at 37 °C. Following extraction, the N-glycans were fluorescently labeled with 2-aminobenzamide (2-AB) (Sigma Aldrich, St. Louis, MO, USA) after 2 h incubation at 65 °C.

The separation of the fluorescently labeled plasma N-glycans was performed using HILIC with an Acquity Ultraperformance Liquid Chromatographic (UPLC) instrument (Waters, Milford, MA, USA) on a Waters BEH Glycan chromatography column (150 × 2.1 mm i.d., 1.7 μm BEH particles) at 25 °C with 100 mM ammonium formate as solvent A (pH 4.4) and acetonitrile as solvent B. A linear gradient of the solvent A (30–47%) at a 0.56 mL/min flow rate for 23 min, and the fluorescence detector set with the excitation wavelength of 250 nm and the emission wavelength of 428 nm, were used to perform the runs. The retention times for individual N-glycans were converted to glucose units using an external standard of hydrolyzed and 2-AB-labeled glucose oligomers for calibration.

The obtained chromatograms were separated into 39 chromatographic peaks for which the major N-glycan structures have been previously assigned [46,83] (Supplementary Table S1). The amount of the N-glycans present in each peak was expressed as a percentage of the total integrated chromatographic area using an automatic method with a traditional integration algorithm and with manual correction afterward to maintain the same intervals of integration for all samples.

### 4.4. Genotyping

The *FUT8* gene-related polymorphisms, rs6573604, rs11621121, rs10483776, and rs4073416, were determined with the TaqMan Genotyping Assay (Applied Biosystems, Foster City, CA, USA) using the Applied Biosystems R 7300 Real-Time PCR System, according to the manufacturer’s protocol. The genotyping was performed in 10 µL reaction volume, which contained around 20 ng of DNA with the thermocycler conditions for the TaqMan Genotyping Assays: 10 min at 95 °C (initial denaturation), 40 cycles of 95 °C for 15 s, and 60 °C for 1 min.

### 4.5. Statistical Analysis

The N-glycan data obtained by UPLC were expressed as percentages of the total area under the curve, after normalization and batch correction, performed to remove the experimental variation in the measurements, as described previously [8]. R Statistics 3.5.1 software was used for the statistical analyses and figure preparation. Haploview 4.2 software [84] was used to determine HWE using the χ^2^-test and LD values between the rs6573604, rs11621121, rs10483776, and rs4073416 polymorphisms based on the confidence interval method [85]. For the haplotype blocks that were in the strong LD (D′ > 0.80), an expectation maximization algorithm integrated into the PLINK 1.07 software [86] was used to assign the most probable haplotype pair for each subject. The frequency of occurrence of the different genotypes, alleles, and haplotypes of the tested polymorphisms in the PTSD patients and control participants was evaluated with the χ^2^ test. Standardized residuals (R) were calculated to determine which parameter contributed the most to the significant differences between the groups [87].

A Kolmogorov–Smirnov test was used to assess the normality of the data distribution for each N-glycan peak. Since the data distribution deviated from normal in most cases, the results were expressed as the median and interquartile range (25th and 75th percentile). The results were presented with the box-plot diagrams, where the central box represented the interquartile range of the age-adjusted percentage of the total glycan peak area, the middle line represented the median, the vertical line extended from the minimum to the maximum value, while separate dots represented the outliers (values lying more than 1.5 box-lengths and less than 3 box-lengths outside of the box). Extreme values (more than 3 box-lengths outside of the box) were excluded from the analyses. Multiple linear regression was used to determine the effect of age, BMI, and diagnosis on plasma N-glycan levels. Since it is known that N-glycans are highly influenced by age [88], and age was a significant predictor in this model, fitting the linear model of each glycan peak depending on age and using the obtained residuals for further analysis was used to correct for this effect [7,8]. OPLS-DA with the age-corrected levels of the plasma N-glycans as variables, and obtained VIP scores and correlation coefficients values—p(corr) for each N-glycan, were used to demonstrate the differences in the N-glycome between the PTSD and control group [89] and all further analyses were performed separately in the control participants and the patients with PTSD.

The differences in the relative abundance of the N-glycan peaks in the participants subdivided according to different the rs6573604, rs11621121, rs10483776, and rs4073416 genotypes (genetic model) or alleles (allelic model) were evaluated using the Kruskal–Wallis ANOVA on ranks with the post hoc Dunn test and the Mann–Whitney U test, respectively. The FDR (Benjamini–Hochberg) method was used to correct the *p*-values for the number of tested N-glycan species. The corrected values of p_BH_ < 0.05 were considered significant.

## Figures and Tables

**Table 1:** Description of the enrolled rs6573604, rs11621121, rs10483776, and rs4073416 polymorphisms located in the *FUT8* gene-related region.

SNP	Position (bp)	Associated Gene	MAF (EU)	MAF	HWE
rs6573604	14:65321223	*PTBP1P*, *MIR4708*	C = 0.185	C = 0.190	***p* = 0.003**
rs11621121	14:65355775	*MIR4708*, *FUT8*	C = 0.410	C = 0.440	*p* = 0.467
rs10483776	14:65448149	*FUT8*	G = 0.176	G = 0.207	*p* = 0.487
rs4073416	14:65792676	*NCOA4P1*, *FUT8*	C = 0.419	C = 0.416	*p* = 0.516

Bp—base pairs, *FUT8*—fucosyltransferases 8, HWE—Hardy–Weinberg equilibrium, MAF—minor allele frequency, MAF (EU)—minor allele frequency in the European population, *MIR4708*—microRNA 4708, *NCOA4P1*—nuclear receptor coactivator 4 pseudogene 1, *PTBP1P*—polypyrimidine tract binding protein 1 pseudogene, SNP—single nucleotide polymorphism. *p*-value denoted in bold is considered statistically significant.

**Table 2:** Distribution of the genotypes and alleles of the rs6573604, rs11621121, rs10483776, and rs4073416 polymorphisms in the control participants and the patients with PTSD.

SNP	Genotype/Allele	Control Participants		Patients with PTSD		Statistics
		N	%	N	%	
rs6573604	CC	20	8.1%	11	3.7%	χ^2^ = 5.861; df = 2; *p* = 0.053
	CT	69	28.1%	75	25.4%	
	TT	157	63.8%	209	70.8%	
	C	109	22.2%	97	16.4%	χ^2^ = 5.682; df = 1; ***p* = 0.017**
	T	383	77.8%	493	83.6%	
rs11621121	CC	51	20.7%	58	19.7%	χ^2^ = 2.571; df = 2; *p* = 0.277
	CT	124	50.4%	133	45.1%	
	TT	71	28.9%	104	35.3%	
	C	226	45.9%	249	42.2%	χ^2^ = 1.517; df = 1; *p* = 0.218
	T	266	54.1%	341	57.8%	
rs10483776	AA	160	65.0%	179	60.7%	χ^2^ = 2.473; df = 2; *p* = 0.290
	AG	75	30.5%	107	36.3%	
	GG	11	4.5%	9	3.1%	
	A	395	80.3%	465	78.8%	χ^2^ = 0.356; df = 1; *p* = 0.551
	G	97	19.7%	125	21.2%	
rs4073416	CC	46	18.7%	43	14.6%	χ^2^ = 2.958; df = 2; *p* = 0.228
	CT	114	46.3%	157	53.2%	
	TT	86	35.0%	95	32.2%	
	C	206	41.9%	243	41.2%	χ^2^ = 0.052; df = 1; *p* = 0.820
	T	286	58.1%	347	58.8%	

Data are presented as total count (N) and frequency (%) and analyzed using the χ^2^-test. *p*-value denoted in bold is considered statistically significant. SNP—single nucleotide polymorphism.

**Table 3:** Distribution of the rs11621121–rs10483776 haplotypes in all enrolled participants, as well as in the participants in the control and the PTSD groups.

Haplotype rs11621121–rs10483776	All Participants		Control Participants		Patients with PTSD	
	N	%	N	%	N	%
TA	601	55.5%	264	53.5%	337	57.1%
CA	259	23.9%	131	26.6%	128	21.7%
CG	216	20.0%	95	19.3%	121	20.5%
TG	6	0.5%	2	0.4%	4	0.7%
Statistics			χ^2^ = 3.853; df = 3; *p* = 0.278			

Data are presented as total count (N) and frequency (%) and analyzed using the χ^2^-test.

**Table 4:** Significant associations of the individual rs6573604, rs11621121, rs10483776, and rs4073416 polymorphisms (genetic and allelic model) as well as the rs11621121–rs10483776 haplotypes with the plasma N-glycan levels in the control participants.

Glycan Peak	Model	rs6573604		rs11621121		rs10483776		rs4073416		Haplo-Type
		H/U *	*p*	H/U *	*p*	H/U *	*p*	H/U *	*p*	
GP08 (A2G2)	Genetic	0.06	0.999	9.06	0.215	2.95	0.593	10.26	0.117	H = 26.75; *p* = 0.101
	Allelic	20754.5	0.978	25454.0	0.059	17330.5	0.333	24352.0	**0.020**	
GP14 (A2G2S1)	Genetic	0.62	0.893	4.61	0.650	5.24	0.475	9.85	0.091	H = 21.39; *p* = 0.351
	Allelic	19956.5	0.726	26820.0	0.254	16446.5	0.202	24472	**0.013**	
GP22 (FA2G2S2)	Genetic	1.56	0.744	21.00	**0.001**	22.05	**<0.001**	14.61	**0.039**	H = 31.41; ***p* < 0.001**
	Allelic	19856.5	0.710	23408.0	**0.001**	13506.5	**<0.001**	23952	**0.016**	
GP29 (FA3G3S3)	Genetic	3.99	0.530	4.07	0.464	12.85	**0.020**	6.99	0.234	H = 28.37; ***p* = 0.020**
	Allelic	18026.5	0.167	27664.0	0.333	14608.5	**0.003**	25248	0.068	
GP31 (FA3G3S3)	Genetic	3.94	0.493	8.69	0.169	24.36	**<0.001**	1.29	0.757	H = 41.19; ***p* < 0.001**
	Allelic	18042.5	0.151	25588.0	0.052	13052.5	**<0.001**	27646	0.397	
GP34 (A4G4S3)	Genetic	6.66	0.281	2.57	0.540	13.53	**0.013**	1.38	0.755	H = 14.39; ***p* = 0.013**
	Allelic	17340.5	0.068	27578.0	0.345	14452.5	**0.002**	28230	0.559	
GP36 (A4G4S4)	Genetic	15.13	**0.020**	4.58	0.563	1.44	0.702	0.41	0.881	H = 23.30; *p* = 0.256
	Allelic	16010.5	**0.003**	26752.0	0.341	18606.5	0.781	28718	0.706	
GP37 (A4G4S4)	Genetic	15.14	**0.013**	0.10	0.978	3.50	0.522	0.18	0.912	H = 35.03; *p* = 0.316
	Allelic	15514.5	**0.001**	29586.0	0.876	16828.5	0.273	28868	0.743	
GP38 (A4G4S4)	Genetic	17.40	**0.007**	0.85	0.728	1.62	0.666	0.23	0.914	H = 33.42; *p* = 0.440
	Allelic	15342.5	**0.001**	28706.0	0.585	17988.5	0.622	29370	0.980	

* Data analyzed using the Kruskal–Wallis ANOVA of ranks for the genetic model and represented with the H value, or the Mann–Whitney U test for the allelic model and represented with the U value. Significant *p*-values (corrected using the Benjamini–Hochberg procedure) are denoted in bold.

**Table 5:** Significant associations of the individual rs6573604, rs11621121, rs10483776, and rs4073416 polymorphisms (genetic and allelic model) as well as the rs11621121–rs10483776 haplotypes with the plasma N-glycan levels in the PTSD patients.

Glycan Peak	Model	rs6573604		rs11621121		rs10483776		rs4073416		Haplo-Type
		H/U *	*p*	H/U *	*p*	H/U *	*p*	H/U *	*p*	
GP16 (FA2G2S1)	Genetic	0.18	0.991	11.51	**0.039**	15.50	**0.017**	6.85	0.322	H = 21.10; ***p* = 0.029**
	Allelic	23364.5	0.908	35666.5	**0.020**	24838.5	0.169	37340.5	0.176	
GP20 (A2G2S2)	Genetic	2.82	0.683	7.82	0.156	6.19	0.160	3.92	0.687	H = 20.13; *p* = 0.109
	Allelic	22042.5	0.794	36544.5	**0.039**	26016.5	0.255	38474.5	0.341	
GP22 (FA2G2S2)	Genetic	0.05	1.000	19.34	**0.002**	12.92	**0.026**	11.30	0.156	H = 29.04; ***p* = 0.002**
	Allelic	23552.5	0.936	33526.5	**0.001**	23454.5	**0.039**	36856.5	0.351	
GP23 (FA2BG2S2)	Genetic	0.94	0.938	12.78	**0.039**	9.19	0.065	7.69	0.273	H = 21.73; ***p* = 0.026**
	Allelic	23606.5	0.865	35466.5	**0.013**	26536.5	0.351	37268.5	0.208	
GP31 (FA3G3S3)	Genetic	2.34	0.674	6.57	0.160	13.75	**0.020**	7.99	0.351	H = 20.77; ***p* = 0.020**
	Allelic	22074.5	0.754	37016.5	**0.045**	23276.5	**0.020**	37588.5	0.195	

* Data analyzed using the Kruskal–Wallis ANOVA of ranks for the genetic model and represented with the H value, or the Mann–Whitney U test for the allelic model and represented with the U value. Significant *p*-values (corrected using the Benjamini–Hochberg procedure) are denoted in bold.

## Data Availability

Data available on request.

## Supplementary Materials

The following supporting information can be downloaded at: https://www.mdpi.com/article/10.3390/ijms24065706/s1, Table S1: Plasma N-glycan peaks separated by HILIC and their dominant composition; Table S2: List of N-glycan peaks with their *p*-values obtained by univariate analysis (corrected with the Benjamini–Hochberg procedure), the correlation coefficients values, and the VIP obtained by the OPLS-DA; Table S3: Associations of the rs6573604 and rs11621121 polymorphisms (genetic and allelic model) with the plasma N-glycan levels in the control participants and the patients with PTSD; Table S4: Associations of the rs10483776 and rs4073416 polymorphisms (genetic and allelic model) with the plasma N-glycan levels in the control participants and the patients with PTSD; Table S5: Associations of the rs11621121–rs10483776 haplotypes with the plasma N-glycan level in the control participants and the patients with PTSD; Figure S1: Direction and size of glycan concentration change in the minor allele carriers compared to carriers of the major allele of (a) rs6573604, (b) rs11621121, (c) rs10483776, and (d) rs4073416 polymorphism in control participants; Figure S2: Direction and size of glycan concentration change in the minor allele carriers compared to carriers of the major allele of (a) rs6573604, (b) rs11621121, (c) rs10483776, and (d) rs4073416 polymorphism in PTSD patients.
