# Supplementary material for: The Association of the Polymorphisms in the FUT8-Related Locus with the Plasma Glycosylation in Post-Traumatic Stress Disorder
Source: Int J Mol Sci. 2023 Mar 16;24(6):5706. doi: 10.3390/ijms24065706 (PMC10056189; doi:10.3390/ijms24065706)
Supplement: Supplementary file 1 [file ijms-24-05706-s001.zip › ijms-2219741-supplementary.pdf]

Supplementary Table S1. The plasma N-glycan peaks separated by HILIC and their dominant composition

Glycan peak	Major glycan structure	Description
**GP01**	FA2	agalactosylated
**GP02**	M5, FA2B	high mannose (M5), agalactosylated with bisecting GlcNAc (FA2B)
**GP03**	A2[6]BG1	monogalactosylated with bisecting GlcNAc
**GP04**	FA2[6]G1	monogalactosylated with core fucose
**GP05**	FA2[3]G1	monogalactosylated with core fucose
**GP06**	FA2[6]BG1	monogalactosylated with core fucose and bisecting GlcNAc
**GP07**	M6	high mannose
**GP08**	A2G2	digalactosylated
**GP09**	A2BG2	digalactosylated with bisecting GlcNAc
**GP10**	FA2G2	digalactosylated with core fucose
**GP11**	FA2BG2	digalactosylated with core fucose and bisecting GlcNAc
**GP12**	A2[3]BG1S(3)1	monogalactosylated with bisecting GlcNAc
**GP13**	FA2[3]G1S(3)1	monogalactosylated and sialylated with core fucose
**GP14**	A2G2S(6)1	digalactosylated and sialylated
**GP15**	A2BG2S1	digalactosylated and sialylated with bisecting GlcNAc
**GP16**	FA2G2S(6)1	digalactosylated and sialylated with core fucose
**GP17**	FA2BG2S(3)1	digalactosylated and sialylated with bisecting GlcNAc and core fucose
**GP18**	A2G2S(3,6)2	digalactosylated and disialylated
**GP19**	M9	high mannose
**GP20**	A2G2S(3,6)2	digalactosylated and disialylated
**GP21**	A2BG2S2	digalactosylated and disialylated with bisecting GlcNAc
**GP22**	FA2G2S(3,6)2	digalactosylated and disialylated with core fucose
**GP23**	FA2BG2S(3,6)2	digalactosylated and disialylated with bisecting GlcNAc and core fucose
**GP24**	A3G3S(3,6)2	trigalactosylated and disialylated
**GP25**	A3BG3S2	trigalactosylated and disialylated with bisecting GlcNAc
**GP26**	A3G3S(3,3)2	trigalactosylated and disialylated
**GP27**	A3G3S(3,3,3)3	trigalactosylated and trisialylated
**GP28**	A3G3S(3,3,6)3	trigalactosylated and trisialylated
**GP29**	FA3G3S(3,3,3)3	trigalactosylated and trisialylated with core fucose
**GP30**	A3G3S(3,3,6)3	trigalactosylated and trisialylated
**GP31**	FA3G3S(3,3,6)3	trigalactosylated and trisialylated with core fucose
**GP32**	A3F1G3S(3,3,3)3	trigalactosylated and trisialylated with antennary fucose
**GP33**	A4G4S(3,3,3)3	tetragalactosylated and trisialylated
**GP34**	A4G4S(3,3,6)3	tetragalactosylated and trisialylated
**GP35**	A4F1G3S(3,3,3)3	tetragalactosylated and trisialylated with antennary fucose
**GP36**	A4G4S(3,3,3,3)4	tetragalactosylated and tetrasialylated
**GP37**	A4G4S(3,3,3,6)4	tetragalactosylated and tetrasialylated
**GP38**	A4G4S(3,6,6,6)4	tetragalactosylated and tetrasialylated
**GP39**	A4F1G4S(3,3,3,6)4	tetragalactosylated and etrasialylated with antennary fucose

Structure abbreviations: all N-glycans have two core N-acetylglucosamines (GlcNAcs); F at the start of the abbreviation indicates core fucose α1-6 linked to the inner GlcNAc; Mx, number (x) of mannose on the core GlcNAcs; Ax, number of antennae (GlcNAc) on the trimannosyl core; B, bisecting GlcNAc linked β1-4 to β1-3 mannose; Gx, number (x) of β1-4 linked galactose on the antenna; [3]G1 and [6]G1 indicates that the galactose is present on the antenna of the α1-3 or α1-6 mannose; F(x), number (x) of fucose linked α1-3 to the antenna GlcNAc; Sx, number (x) of sialic acids linked to galactose; the numbers 3 or 6 or in parentheses after S indicate whether the sialic acid is in a α2-3 or α2-6 linkage. If there is no linkage number, the exact link is unknown.

Supplementary Table S2. The list of the N-glycan peaks with their p-values (p_{BH}) obtained by the univariate analysis (corrected with the Benjamini-Hochberg procedure), the correlation coefficients values (p(corr)), and variable importance in the projection (VIP) obtained by the Supervised Orthogonal Partial Least Square – Discriminant Analysis (OPLS-DA).

Glycan peak	p_{BH}	p(corr)	VIP
GP01	0.857	-0.084	0.396
GP02	0.962	0.092	0.757
GP03	0.526	0.253	0.773
GP04	**0.039**	**-0.449**	**1.207**
GP05	**0.010**	**-0.527**	**1.348**
GP06	0.459	-0.116	0.869
GP07	0.009	-0.071	0.750
GP08	0.011	0.330	0.799
GP09	0.839	0.133	0.885
GP10	**0.016**	**-0.461**	**1.215**
GP11	0.078	-0.055	0.921
GP12	0.700	-0.040	0.437
GP13	0.117	-0.354	1.040
GP14	0.002	0.375	0.937
GP15	0.649	0.131	0.769
GP16	**0.008**	**-0.424**	**1.306**
GP17	0.249	0.022	0.819
GP18	0.343	-0.262	0.681
GP19	**0.002**	**-0.455**	**1.102**
GP20	0.924	0.063	0.776
GP21	0.903	0.195	0.488
GP22	0.039	-0.349	0.839
GP23	0.577	-0.023	0.678
GP24	0.335	-0.126	0.486
GP25	0.963	0.050	0.524
GP26	0.687	0.015	0.534
GP27	**0.001**	**0.700**	**1.715**
GP28	0.588	-0.311	0.868
GP29	0.140	-0.227	0.726
GP30	0.919	-0.183	0.722
GP31	0.095	-0.386	1.089
GP32	**0.050**	**0.377**	**1.112**
GP33	**0.001**	**0.688**	**1.699**
GP34	0.259	0.092	0.852
GP35	**0.015**	**0.502**	**1.302**
GP36	**<0.001**	**0.605**	**1.590**
GP37	0.348	0.010	0.515
GP38	**<0.001**	**0.437**	**1.250**
GP39	**<0.001**	**0.693**	**1.706**

Variables with $p_{BH} \leq 0.05$, absolute p(corr) ≥ 0.30, and VIP > 1.000 were considered the significant contributors in discriminating the diagnostic groups and are denoted in bold. Negative p(corr) indicates diminished glycan concentrations in the plasma of PTSD patients versus control participants, whereas positive p(corr) values indicate increased glycan concentrations in PTSD patients.

Supplementary Table S3. The associations of the rs6573604 and rs11621121 polymorphisms (genetic and allelic model) with the plasma N-glycan levels in the control participants and the patients with PTSD.

Glycan peak	Group	rs6573604						rs11621121					
		**Genetic model**			**Allelic model**			**Genetic model**			**Allelic model**		
		H	p	p_{BH}	U	p	p_{BH}	H	p	p_{BH}	U	p	p_{BH}
**GP01**	Control	1.566	0.457	0.775	20518.5	0.786	0.876	0.930	0.628	0.742	28566.0	0.342	0.534
	PTSD	0.480	0.786	0.929	23138.5	0.615	0.923	0.836	0.658	0.778	40804.5	0.420	0.546
**GP02**	Control	1.278	0.528	0.735	20120.5	0.565	0.787	2.315	0.314	0.510	28078.0	0.208	0.406
	PTSD	1.551	0.461	0.782	23344.5	0.712	0.926	1.871	0.392	0.637	39552.5	0.156	0.304
**GP03**	Control	5.631	0.060	0.334	18534.5	0.074	0.241	2.640	0.267	0.548	27566.0	0.113	0.367
	PTSD	3.388	0.184	0.652	21940.5	0.199	0.776	0.156	0.925	0.925	42282.5	0.933	0.933
**GP04**	Control	1.997	0.368	0.718	20160.5	0.586	0.737	6.027	0.049	0.478	26852.0	0.041	0.200
	PTSD	1.128	0.569	0.888	22270.5	0.285	0.695	0.751	0.687	0.788	40668.5	0.382	0.514
**GP05**	Control	1.450	0.484	0.726	20092.5	0.551	0.796	1.109	0.574	0.700	28440.0	0.303	0.514
	PTSD	3.373	0.185	0.601	22926.5	0.521	0.813	0.343	0.842	0.888	41230.5	0.549	0.630
**GP06**	Control	2.510	0.285	0.654	18780.5	0.110	0.268	1.629	0.443	0.596	28188.0	0.234	0.415
	PTSD	2.718	0.257	0.626	21776.5	0.164	0.711	7.353	0.025	0.139	37042.5	0.008	0.052
**GP07**	Control	0.286	0.867	0.939	20124.5	0.567	0.763	3.157	0.206	0.502	27666.0	0.128	0.357
	PTSD	3.765	0.152	0.741	23338.5	0.709	0.953	3.153	0.207	0.449	39672.5	0.174	0.308
**GP08**	Control	0.056	0.973	0.999	20754.5	0.928	0.978	9.067	0.011	0.215	25454.0	0.003	0.059
	PTSD	3.143	0.208	0.624	21110.5	0.068	0.332	5.332	0.070	0.248	38938.5	0.086	0.224
**GP09**	Control	2.193	0.334	0.686	20334.5	0.681	0.781	2.468	0.291	0.516	27882.0	0.166	0.341
	PTSD	4.004	0.135	1.053	23158.5	0.624	0.901	2.856	0.240	0.468	39076.5	0.099	0.241
**GP10**	Control	1.907	0.385	0.715	19020.5	0.157	0.360	4.097	0.129	0.503	27860.0	0.162	0.351
	PTSD	0.107	0.948	0.999	23438.5	0.758	0.924	1.449	0.484	0.699	40448.5	0.327	0.455
**GP11**	Control	1.135	0.567	0.763	19508.5	0.297	0.610	0.923	0.630	0.723	29108.0	0.546	0.761
	PTSD	3.837	0.147	0.819	22398.5	0.325	0.704	5.560	0.062	0.242	37500.5	0.015	0.059
**GP12**	Control	1.748	0.417	0.739	19690.5	0.366	0.714	0.088	0.957	0.957	29596.0	0.769	0.857
	PTSD	0.448	0.799	0.917	23572.5	0.826	0.920	0.338	0.845	0.867	41502.5	0.642	0.696
**GP13**	Control	0.492	0.782	0.924	19874.5	0.446	0.696	1.488	0.475	0.598	29310.0	0.634	0.824
	PTSD	0.896	0.639	0.923	22522.5	0.366	0.751	5.167	0.076	0.228	37634.5	0.018	0.059
**GP14**	Control	0.621	0.733	0.893	19956.5	0.484	0.726	4.610	0.100	0.650	26820.0	0.039	0.254
	PTSD	5.176	0.075	0.731	20324.5	0.019	0.185	7.801	0.020	0.195	36918.5	0.007	0.055
**GP15**	Control	0.343	0.843	0.939	20200.5	0.607	0.717	4.482	0.106	0.517	26818.0	0.039	0.217
	PTSD	2.347	0.309	0.709	22288.5	0.291	0.668	2.447	0.294	0.521	39134.5	0.104	0.239
**GP16**	Control	2.709	0.258	0.629	18734.5	0.102	0.265	3.169	0.205	0.533	28132.0	0.220	0.409
	PTSD	0.179	0.915	0.991	23364.5	0.722	0.908	11.512	0.003	**0.039**	35666.5	0.001	**0.020**
**GP17**	Control	0.123	0.941	0.992	20770.5	0.937	0.962	4.371	0.112	0.485	26798.0	0.038	0.296
	PTSD	2.053	0.358	0.698	22696.5	0.429	0.761	6.680	0.035	0.171	37424.5	0.014	0.068
**GP18**	Control	0.445	0.801	0.919	20142.5	0.577	0.750	2.436	0.296	0.502	29370.0	0.662	0.807
	PTSD	6.913	0.032	0.416	20228.5	0.016	0.208	1.292	0.524	0.705	41508.5	0.644	0.679
**GP19**	Control	3.829	0.147	0.478	18164.5	0.039	0.169	3.240	0.198	0.552	27456.0	0.098	0.347
	PTSD	0.409	0.815	0.908	23588.5	0.834	0.904	2.777	0.249	0.462	41054.5	0.494	0.602
**GP20**	Control	4.085	0.130	0.563	18362.5	0.055	0.195	2.224	0.329	0.513	29748.0	0.844	0.890
	PTSD	2.816	0.245	0.683	22042.5	0.224	0.794	7.233	0.027	0.156	36544.5	0.005	**0.049**
**GP21**	Control	8.104	0.017	0.166	20168.5	0.590	0.719	3.696	0.158	0.474	27106.0	0.060	0.234
	PTSD	1.808	0.405	0.752	22764.5	0.455	0.772	0.470	0.791	0.857	41118.5	0.514	0.607
**GP22**	Control	1.563	0.458	0.744	19856.5	0.437	0.710	21.000	<0.001	**0.001**	23408.0	<0.001	**0.001**
	PTSD	0.054	0.974	1.000	23552.5	0.816	0.936	19.336	<0.001	**0.002**	33526.5	<0.001	**0.001**
**GP23**	Control	0.037	0.981	0.981	20638.5	0.858	0.930	5.494	0.064	0.499	30006.0	0.974	1.000
	PTSD	0.939	0.625	0.938	23606.5	0.843	0.865	12.784	0.002	**0.039**	35466.5	0.001	**0.013**
**GP24**	Control	2.807	0.246	0.640	18586.5	0.081	0.243	2.726	0.256	0.587	29694.0	0.817	0.885
	PTSD	1.173	0.556	0.904	22202.5	0.266	0.741	3.312	0.191	0.466	39296.5	0.123	0.267
**GP25**	Control	2.331	0.312	0.676	18728.5	0.101	0.281	2.031	0.362	0.523	29358.0	0.656	0.825

Glycan peak	Group	rs6573604						rs11621121					
		Genetic model			Allelic model			Genetic model			Allelic model		
		H	p	p_{BH}	U	p	p_{BH}	H	p	p_{BH}	U	p	p_{BH}
	PTSD	6.996	0.030	0.585	19704.5	0.006	0.234	3.326	0.190	0.494	39522.5	0.152	0.312
**GP26**	Control	1.097	0.578	0.751	19744.5	0.389	0.690	2.511	0.285	0.529	28838.0	0.438	0.633
	PTSD	2.785	0.248	0.645	22232.5	0.274	0.712	4.870	0.088	0.245	37846.5	0.024	0.072
**GP27**	Control	1.000	0.606	0.762	20838.5	0.979	0.979	0.199	0.905	0.954	29402.0	0.676	0.799
	PTSD	0.535	0.765	0.962	23526.5	0.802	0.948	2.215	0.330	0.560	39884.5	0.209	0.354
**GP28**	Control	3.167	0.205	0.571	18332.5	0.052	0.203	2.034	0.362	0.504	28456.0	0.308	0.501
	PTSD	3.438	0.179	0.698	20964.5	0.055	0.358	0.546	0.761	0.848	41616.5	0.682	0.700
**GP29**	Control	3.986	0.136	0.530	18026.5	0.030	0.167	4.068	0.131	0.464	27664.0	0.128	0.333
	PTSD	7.226	0.027	1.000	19704.5	0.006	0.117	0.954	0.621	0.781	41356.5	0.591	0.659
**GP30**	Control	5.343	0.069	0.336	17752.5	0.017	0.111	1.536	0.464	0.603	30018.0	0.980	0.980
	PTSD	0.782	0.676	0.942	22652.5	0.412	0.803	3.093	0.213	0.437	38896.5	0.082	0.228
**GP31**	Control	3.945	0.139	0.493	18042.5	0.031	0.151	8.694	0.013	0.169	25588.0	0.004	0.052
	PTSD	2.337	0.311	0.674	22074.5	0.232	0.754	6.571	0.037	0.160	37016.5	0.008	**0.045**
**GP32**	Control	3.642	0.162	0.486	19726.5	0.381	0.708	3.927	0.140	0.455	27034.0	0.054	0.234
	PTSD	3.645	0.162	0.702	20904.5	0.050	0.390	5.311	0.070	0.228	37562.5	0.017	0.060
**GP33**	Control	1.459	0.482	0.752	19818.5	0.420	0.712	0.348	0.840	0.910	29212.0	0.590	0.793
	PTSD	0.654	0.721	0.937	23588.5	0.834	0.879	1.660	0.436	0.654	40166.5	0.263	0.395
**GP34**	Control	6.657	0.036	0.281	17340.5	0.007	0.068	2.569	0.277	0.540	27578.0	0.115	0.345
	PTSD	2.070	0.355	0.729	22688.5	0.426	0.791	1.320	0.517	0.720	40026.5	0.235	0.382
**GP35**	Control	1.444	0.486	0.702	19230.5	0.210	0.455	2.669	0.263	0.570	27672.0	0.129	0.314
	PTSD	0.495	0.781	0.952	22836.5	0.484	0.787	1.244	0.537	0.698	40096.5	0.249	0.388
**GP36**	Control	15.133	0.001	**0.020**	16010.5	<0.001	**0.003**	4.583	0.101	0.563	26752.0	0.035	0.341
	PTSD	0.686	0.710	0.955	23168.5	0.629	0.876	7.540	0.023	0.150	37424.5	0.014	0.061
**GP37**	Control	15.139	0.001	**0.013**	15514.5	<0.001	**0.001**	0.096	0.953	0.978	29586.0	0.764	0.876
	PTSD	3.877	0.144	0.936	21044.5	0.062	0.345	1.705	0.426	0.665	40344.5	0.302	0.436
**GP38**	Control	17.400	<0.001	**0.007**	15342.5	<0.001	**0.001**	0.853	0.653	0.728	28706.0	0.390	0.585
	PTSD	1.702	0.427	0.757	22152.5	0.252	0.756	3.287	0.193	0.443	39568.5	0.158	0.293
**GP39**	Control	5.976	0.050	0.325	17626.5	0.013	0.101	2.196	0.334	0.501	27760.0	0.144	0.330
	PTSD	0.004	0.998	0.998	23874.5	0.981	0.981	0.931	0.628	0.765	41018.5	0.483	0.608

The data are represented as the Kruskal-Wallis H value or the Mann-Whitney U value, the original p-value, and the p-value corrected with the Benjamini-Hochberg procedure (p_{BH}). Significant p_{BH} are denoted in bold.

Supplementary Table S4. The associations of the rs10483776 and rs4073416 polymorphisms (genetic and allelic model) with the plasma N-glycan levels in the control participants and the patients with PTSD.

**Glycan peak**	**Group**	**rs10483776**						**rs4073416**					
		**Genetic model**			**Allelic model**			**Genetic model**			**Allelic model**		
		H	p	pBH	U	p	pBH	H	p	pBH	U	p	pBH
**GP01**	Control	2.266	0.322	0.571	17806.5	0.282	0.611	1.984	0.371	0.629	27528.0	0.215	0.399
	PTSD	0.476	0.788	0.878	27984.5	0.524	0.705	0.419	0.811	0.904	41838.5	0.874	0.921
**GP02**	Control	0.170	0.918	0.968	18746.5	0.743	0.828	3.781	0.151	0.535	27896.0	0.315	0.473
	PTSD	2.669	0.263	0.466	26576.5	0.142	0.346	2.443	0.295	0.677	39138.5	0.138	0.449
**GP03**	Control	0.081	0.960	0.985	18794.5	0.772	0.814	5.141	0.076	0.329	27312.0	0.168	0.385
	PTSD	1.991	0.370	0.601	27324.5	0.304	0.515	2.189	0.335	0.622	39536.5	0.198	0.429
**GP04**	Control	2.321	0.313	0.610	17266.5	0.132	0.322	5.919	0.052	0.338	25580.0	0.013	0.101
	PTSD	1.064	0.588	0.791	28396.5	0.694	0.820	1.655	0.437	0.710	41536.5	0.759	0.987
**GP05**	Control	1.789	0.409	0.638	17826.5	0.289	0.593	2.498	0.287	0.533	27430.0	0.192	0.394
	PTSD	0.310	0.856	0.927	28698.5	0.830	0.925	0.107	0.948	0.948	41694.5	0.819	0.913
**GP06**	Control	2.488	0.288	0.591	18024.5	0.366	0.595	1.500	0.472	0.736	27584.0	0.228	0.387
	PTSD	5.048	0.080	0.240	25536.5	0.037	0.289	6.773	0.034	0.265	37214.5	0.015	0.293
**GP07**	Control	2.757	0.252	0.578	17122.5	0.105	0.315	5.384	0.068	0.332	25762.0	0.018	0.117
	PTSD	4.801	0.091	0.237	26324.5	0.106	0.295	0.794	0.672	0.904	41200.5	0.638	0.889
**GP08**	Control	2.954	0.228	0.593	17330.5	0.145	0.333	10.262	0.006	0.117	24352.0	0.001	**0.020**
	PTSD	0.501	0.779	0.894	27942.5	0.508	0.708	3.541	0.170	0.663	42126.5	0.987	0.987
**GP09**	Control	0.652	0.722	0.828	18380.5	0.536	0.697	3.391	0.183	0.446	27246.0	0.155	0.378
	PTSD	5.688	0.058	0.189	25820.5	0.055	0.306	3.098	0.212	0.591	38972.5	0.118	0.418
**GP10**	Control	3.104	0.212	0.591	17948.5	0.335	0.653	3.314	0.191	0.438	26902.0	0.100	0.325
	PTSD	1.822	0.402	0.627	27732.5	0.432	0.648	1.472	0.479	0.719	39958.5	0.280	0.475
**GP11**	Control	0.948	0.623	0.784	18276.5	0.483	0.650	0.445	0.801	0.893	28572.0	0.569	0.672
	PTSD	9.244	0.010	0.078	25878.5	0.060	0.234	3.376	0.185	0.555	38664.5	0.086	0.335
**GP12**	Control	0.271	0.873	0.946	18492.5	0.596	0.726	5.452	0.065	0.362	26056.0	0.029	0.141
	PTSD	0.922	0.631	0.794	28232.5	0.624	0.785	0.357	0.836	0.906	41310.5	0.677	0.910
**GP13**	Control	2.316	0.314	0.583	17230.5	0.125	0.325	7.641	0.022	0.215	27376.0	0.181	0.392
	PTSD	2.709	0.258	0.479	27046.5	0.233	0.478	2.401	0.301	0.652	39160.5	0.141	0.423
**GP14**	Control	5.245	0.073	0.475	16446.5	0.031	0.202	9.849	0.007	0.091	24472.0	0.001	**0.013**
	PTSD	4.174	0.124	0.269	25758.5	0.051	0.332	3.677	0.159	0.689	39444.5	0.183	0.446
**GP15**	Control	0.966	0.617	0.802	18434.5	0.564	0.710	1.728	0.421	0.684	27560.0	0.222	0.394
	PTSD	6.675	0.036	0.156	27052.5	0.235	0.458	4.197	0.123	0.685	38234.5	0.054	0.301
**GP16**	Control	1.831	0.400	0.650	18098.5	0.399	0.599	3.689	0.158	0.474	27496.0	0.207	0.404
	PTSD	15.503	<0.001	**0.017**	24838.5	0.013	0.169	6.853	0.033	0.322	37340.5	0.018	0.176
**GP17**	Control	2.928	0.231	0.563	16988.5	0.084	0.298	3.308	0.191	0.414	26590.0	0.065	0.282
	PTSD	9.725	0.008	0.078	26748.5	0.171	0.392	3.425	0.180	0.585	38640.5	0.084	0.364
**GP18**	Control	1.349	0.51	0.710	17966.5	0.342	0.635	3.692	0.158	0.440	28566.0	0.566	0.690
	PTSD	1.279	0.527	0.734	28146.5	0.588	0.764	0.139	0.933	0.983	41676.5	0.812	0.931
**GP19**	Control	2.001	0.368	0.624	18748.5	0.744	0.806	3.720	0.156	0.507	27010.0	0.116	0.323
	PTSD	0.168	0.92	0.970	28564.5	0.768	0.881	0.695	0.706	0.860	41592.5	0.780	0.951
**GP20**	Control	3.673	0.159	0.517	16706.5	0.051	0.249	3.679	0.159	0.413	26926.0	0.104	0.312
	PTSD	6.194	0.045	0.160	26016.5	0.072	0.255	3.916	0.141	0.687	38474.5	0.070	0.341
**GP21**	Control	2.755	0.252	0.546	18164.5	0.429	0.620	0.452	0.798	0.915	28716.0	0.633	0.726
	PTSD	1.517	0.468	0.702	27356.5	0.313	0.509	0.492	0.782	0.897	42036.5	0.951	0.976
**GP22**	Control	22.050	<0.001	**<0.001**	13506.5	<0.001	**<0.001**	14.606	0.001	**0.039**	23952.0	<0.001	**0.016**
	PTSD	12.924	0.002	**0.026**	23454.5	0.001	**0.039**	11.303	0.004	0.156	36856.5	0.009	0.351
**GP23**	Control	0.522	0.770	0.858	18936.5	0.860	0.883	1.022	0.600	0.807	28022.0	0.356	0.514
	PTSD	9.194	0.010	0.065	26536.5	0.135	0.351	7.693	0.021	0.273	37268.5	0.016	0.208
**GP24**	Control	1.147	0.563	0.757	18058.5	0.381	0.594	1.105	0.576	0.802	27886.0	0.312	0.487
	PTSD	0.031	0.985	0.985	28978.5	0.96	0.960	1.483	0.476	0.743	39806.5	0.248	0.484
**GP25**	Control	0.708	0.702	0.830	18654.5	0.688	0.789	0.934	0.627	0.815	28162.0	0.405	0.564

Glycan peak	Group	rs10483776						rs4073416					
		Genetic model			Allelic model			Genetic model			Allelic model		
		H	p	pBH	U	p	pBH	H	p	pBH	U	p	pBH
	PTSD	2.275	0.321	0.544	26940.5	0.21	0.455	0.741	0.690	0.868	40494.5	0.414	0.621
**GP26**	Control	0.006	0.997	0.997	19126.5	0.98	0.980	2.918	0.233	0.454	26736.0	0.080	0.312
	PTSD	1.408	0.495	0.715	27608.5	0.39	0.608	2.381	0.304	0.624	39878.5	0.263	0.488
**GP27**	Control	5.551	0.062	0.484	17038.5	0.091	0.296	0.822	0.663	0.834	28166.0	0.406	0.546
	PTSD	6.987	0.03	0.146	25466.5	0.034	0.332	3.531	0.171	0.606	39252.5	0.154	0.429
**GP28**	Control	4.464	0.107	0.464	16458.5	0.031	0.173	0.257	0.879	0.927	29426.0	0.984	0.984
	PTSD	0.663	0.718	0.875	27750.5	0.438	0.633	1.235	0.539	0.751	41572.5	0.773	0.972
**GP29**	Control	12.852	0.002	**0.020**	14608.5	<0.001	**0.003**	6.997	0.030	0.234	25248.0	0.007	0.068
	PTSD	1.056	0.59	0.767	28820.5	0.886	0.909	0.597	0.742	0.877	41592.5	0.780	0.922
**GP30**	Control	0.830	0.660	0.804	18018.5	0.364	0.617	2.410	0.300	0.532	27146.0	0.137	0.356
	PTSD	0.051	0.975	1.001	28774.5	0.865	0.937	1.468	0.480	0.693	40226.5	0.343	0.535
**GP31**	Control	24.363	<0.001	**<0.001**	13052.5	<0.001	**<0.001**	1.293	0.524	0.757	27646.0	0.244	0.397
	PTSD	13.755	0.001	**0.020**	23276.5	0.001	**0.020**	7.989	0.018	0.351	37588.5	0.025	0.195
**GP32**	Control	4.618	0.099	0.483	16420.5	0.029	0.226	4.824	0.090	0.351	25960.0	0.025	0.139
	PTSD	3.972	0.137	0.281	26062.5	0.076	0.247	2.194	0.334	0.651	39292.5	0.159	0.413
**GP33**	Control	4.868	0.088	0.490	17152.5	0.110	0.306	0.723	0.697	0.849	28394.0	0.494	0.621
	PTSD	6.466	0.039	0.152	25860.5	0.058	0.251	2.898	0.235	0.611	39444.5	0.183	0.420
**GP34**	Control	13.535	0.001	**0.013**	14452.5	<0.001	**0.002**	1.376	0.503	0.755	28230.0	0.430	0.559
	PTSD	4.603	0.100	0.229	26212.5	0.092	0.276	2.775	0.250	0.609	39938.5	0.276	0.489
**GP35**	Control	3.882	0.144	0.511	16942.5	0.077	0.300	3.114	0.211	0.433	26730.0	0.080	0.284
	PTSD	3.324	0.19	0.371	28774.5	0.865	0.912	0.119	0.942	0.967	41776.5	0.851	0.922
**GP36**	Control	1.444	0.486	0.702	18606.5	0.661	0.781	0.414	0.813	0.881	28718.0	0.634	0.706
	PTSD	8.367	0.015	0.084	25824.5	0.056	0.273	4.843	0.089	0.579	37934.5	0.038	0.247
**GP37**	Control	3.496	0.174	0.522	16828.5	0.063	0.273	0.185	0.912	0.912	28868.0	0.705	0.743
	PTSD	0.568	0.753	0.890	28290.5	0.648	0.790	0.752	0.687	0.893	41074.5	0.594	0.858
**GP38**	Control	1.622	0.444	0.666	17988.5	0.351	0.622	0.231	0.891	0.914	29370.0	0.955	0.980
	PTSD	4.700	0.095	0.232	27190.5	0.269	0.500	1.897	0.387	0.656	39558.5	0.202	0.415
**GP39**	Control	4.202	0.122	0.476	18268.5	0.479	0.667	0.511	0.775	0.916	28724.0	0.637	0.690
	PTSD	5.061	0.08	0.223	27216.5	0.275	0.488	2.064	0.356	0.631	40196.5	0.335	0.544

The data are represented as the Kruskal-Wallis H value or the Mann-Whitney U value, the original p-value, and the p-value corrected with the Benjamini-Hochberg procedure (p_{BH}). Significant p_{BH} are denoted in bold.

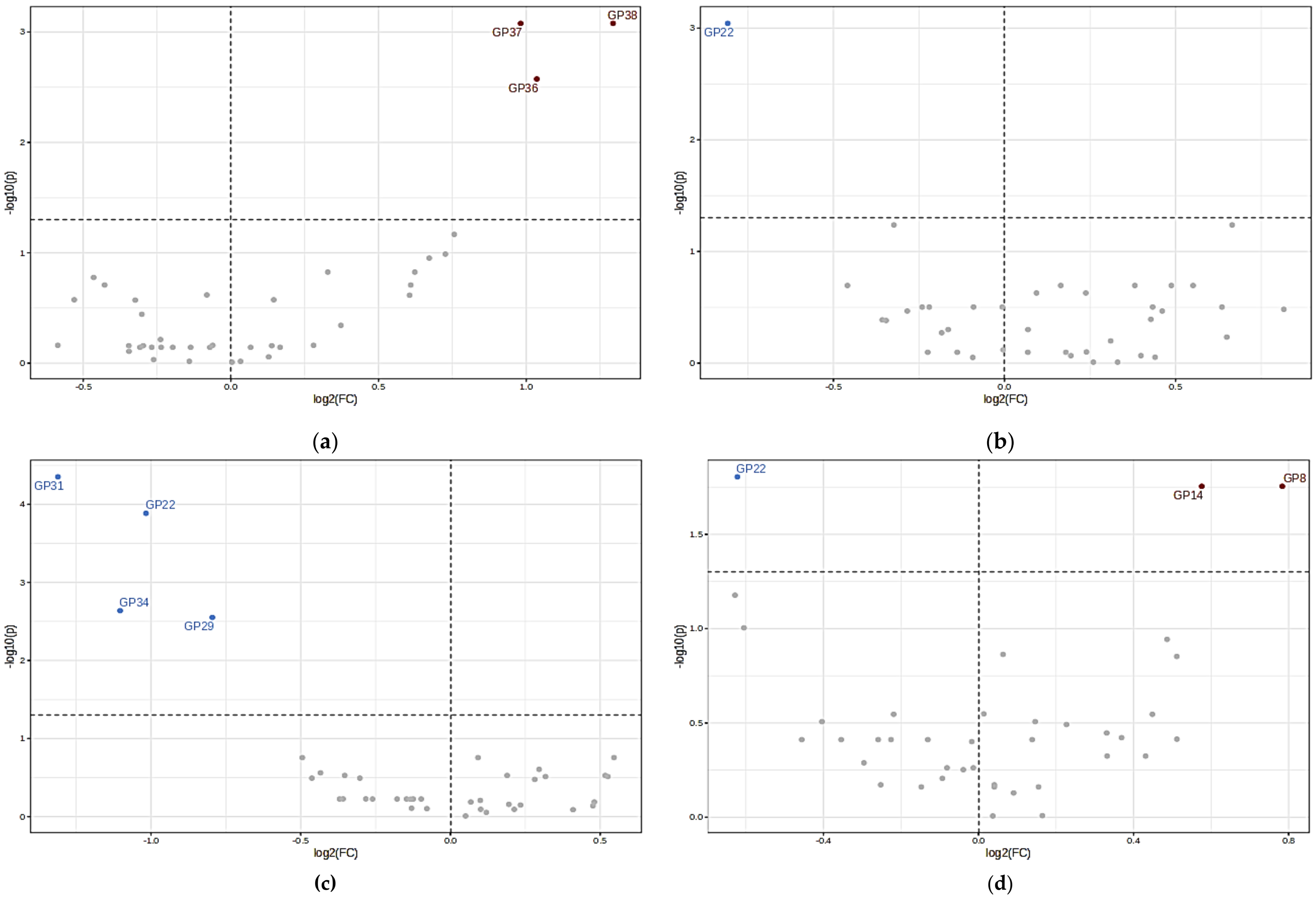

Supplementary Figure S1. Direction and size of glycan concentration change in the minor allele carriers compared to carriers of the major allele of **(a)** rs6573604, **(b)** rs11621121, **(c)** rs10483776, and **(d)** rs4073416 polymorphism in control participants. Significantly increased or decreased glycan peaks are labeled with red or blue circles, respectively, while non-significant glycan peaks are represented with grey circles. log2(FC) = logarithm of fold change; -log10(p) = negative logarithm of FDR corrected p-value (Benjamini-Hochberg method).

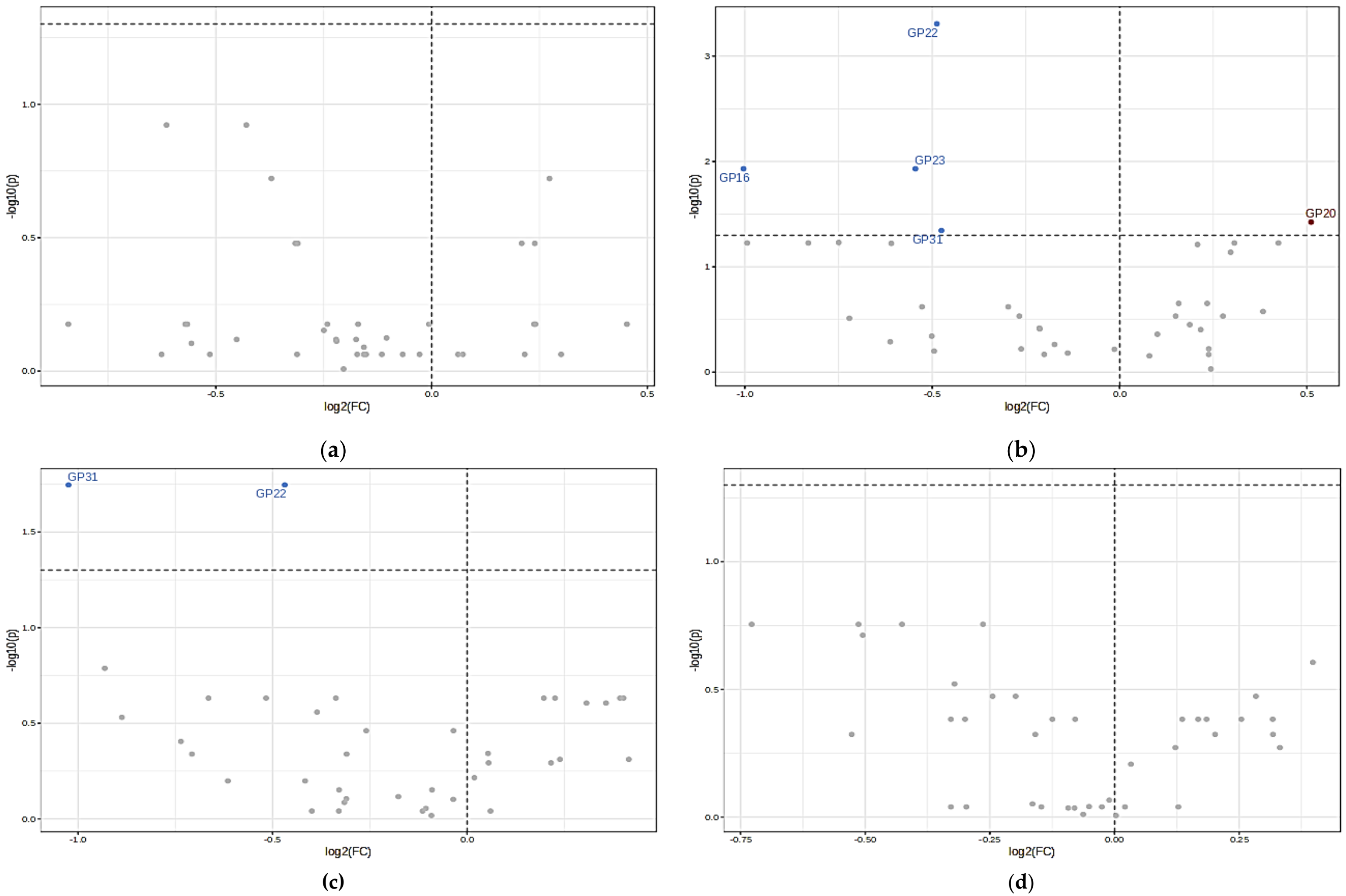

Supplementary Figure S2. Direction and size of glycan concentration change in the minor allele carriers compared to carriers of the major allele of **(a)** rs6573604, **(b)** rs11621121, **(c)** rs10483776, and **(d)** rs4073416 polymorphism in PTSD patients. Significantly increased or decreased glycan peaks are labeled with red or blue circles, respectively, while non-significant glycan peaks are represented with grey circles. log2(FC) = logarithm of fold change; -log10(p) = negative logarithm of FDR corrected p-value (Benjamini-Hochberg method).

Supplementary Table S5. The associations of the rs11621121-rs10483776 haplotypes with the plasma N-glycan levels in the control participants and the patients with PTSD.

Glycan peak	Control participants			Patients with PTSD		
	H	p	p_{BH}	H	p	p_{BH}
**GP01**	1.300	0.522	0.617	1.670	0.434	0.605
**GP02**	1.534	0.465	0.585	3.069	0.216	0.401
**GP03**	4.912	0.086	0.280	1.565	0.457	0.594
**GP04**	4.479	0.107	0.261	2.249	0.325	0.507
**GP05**	1.471	0.479	0.584	0.866	0.649	0.723
**GP06**	1.534	0.464	0.603	7.470	0.024	0.134
**GP07**	3.306	0.191	0.372	2.752	0.253	0.429
**GP08**	8.654	0.013	0.101	4.056	0.132	0.343
**GP09**	2.180	0.336	0.570	3.806	0.149	0.363
**GP10**	2.049	0.359	0.560	1.070	0.586	0.672
**GP11**	0.516	0.773	0.815	6.447	0.040	0.173
**GP12**	0.243	0.886	0.909	0.396	0.820	0.842
**GP13**	2.054	0.358	0.582	6.453	0.040	0.156
**GP14**	5.840	0.054	0.351	8.159	0.017	0.111
**GP15**	5.010	0.082	0.291	3.017	0.221	0.392
**GP16**	1.569	0.456	0.635	11.877	0.003	**0.029**
**GP17**	4.907	0.086	0.258	6.927	0.031	0.151
**GP18**	0.843	0.656	0.731	1.626	0.444	0.597
**GP19**	5.335	0.069	0.384	0.402	0.818	0.862
**GP20**	4.752	0.093	0.242	8.528	0.014	0.109
**GP21**	3.546	0.170	0.368	0.806	0.668	0.724
**GP22**	25.182	<0.001	**<0.001**	20.069	<0.001	**0.002**
**GP23**	0.049	0.976	0.976	12.508	0.002	**0.026**
**GP24**	1.700	0.427	0.617	3.640	0.162	0.372
**GP25**	0.652	0.722	0.782	2.328	0.312	0.507
**GP26**	1.250	0.535	0.614	5.245	0.073	0.219
**GP27**	3.379	0.185	0.380	4.342	0.114	0.318
**GP28**	5.022	0.081	0.351	1.774	0.412	0.595
**GP29**	12.000	0.002	**0.020**	0.307	0.858	0.858
**GP30**	1.549	0.461	0.620	3.584	0.167	0.343
**GP31**	24.234	<0.001	**<0.001**	13.471	0.001	**0.020**
**GP32**	5.325	0.070	0.341	5.924	0.052	0.169
**GP33**	2.807	0.246	0.436	3.605	0.165	0.358
**GP34**	14.390	0.001	**0.013**	3.149	0.207	0.404
**GP35**	3.618	0.164	0.376	1.355	0.508	0.639
**GP36**	4.764	0.092	0.256	6.432	0.040	0.142
**GP37**	5.020	0.081	0.316	1.124	0.570	0.674
**GP38**	2.883	0.237	0.440	2.120	0.346	0.519
**GP39**	2.050	0.359	0.539	1.276	0.528	0.644

The data are represented as the Kruskal-Wallis H value, the original p-value, and the p-value corrected with the Benjamini-Hochberg procedure (p_{BH}). Significant p_{BH} values are denoted in bold.
